# Knowledge and perception of biosimilars in ambulatory care: a survey among Belgian community pharmacists and physicians

**DOI:** 10.1186/s40545-021-00330-x

**Published:** 2021-06-22

**Authors:** Liese Barbier, Yannick Vandenplas, Steven Simoens, Paul Declerck, Arnold G. Vulto, Isabelle Huys

**Affiliations:** 1 Clinical Pharmacology and Pharmacotherapy, Department of Pharmaceutical and Pharmacological Sciences, KU Leuven, Herestraat 49, Box 521, 3000 Leuven, Belgium; 2 Hospital Pharmacy, Erasmus University Medical Center, Box 2040, 3000 CA Rotterdam, The Netherlands

**Keywords:** Biosimilar, Biologic, Community pharmacist, Physician, Healthcare professional, Primary care, Ambulatory care, Interchangeability, Pharmacy substitution

## Abstract

**Background:**

With the approval of biosimilars for subcutaneously administered products, such as adalimumab, etanercept and insulin, biosimilars become increasingly available in ambulatory care. Little is known about the knowledge and attitudes of healthcare providers who are in charge of dispensing and prescribing biosimilars in this context. This study aims to assess the knowledge and perception about biosimilars among community pharmacists and physicians.

**Methods:**

Belgian community pharmacists (*n* = 177) and physicians (*n* = 30) were surveyed on their knowledge, experience with dispensing/prescribing biologicals including biosimilars, perception regarding interchangeability, switching and substitution and informational and educational needs. Descriptive and statistical analyses were performed.

**Results:**

Only 32% of community pharmacists and 52% of physicians had yet dispensed/prescribed a biosimilar. Approximately 35% of community pharmacists felt insufficiently trained to counsel patients with biosimilar therapy, which was significantly higher compared to their self-assessed competence to counsel patients with biological therapy in general (*p* = 0.023). Community pharmacists experienced questions about similarity between reference products and biosimilars (47%) and their interchangeability (42%). Over 40% of physicians found patient uncertainty about efficacy and safety challenging when prescribing biosimilars. A similar proportion of physicians would only prescribe a biosimilar in indications for which the biosimilar has been tested clinically. The majority of pharmacists (58%) was in favor of substitution of biologicals, on the condition that the prescriber would be contacted. Also over 40% of physicians was open to this approach in case of substitution. Educational support, budget for additional staff and transparency about savings were considered suitable stimuli to incentivize biosimilar use. The need for information about biologicals including biosimilars was nearly unanimous among community pharmacists. Also 67% of physicians requested more information. Both community pharmacists and physicians preferred to be informed by their respective professional associations.

**Conclusions:**

This study showed a substantial need for targeted educational measures to increase the knowledge and confidence about both biological medicines in general and biosimilars in particular among Belgian community pharmacists and physicians. The results may inform educational and policy measures to stimulate biosimilar use in ambulatory care.

**Supplementary Information:**

The online version contains supplementary material available at 10.1186/s40545-021-00330-x.

## Background

Biological medicines have substantially altered the treatment pathway of several chronic and life-threatening diseases, positively affecting the life of many patients. The use and success of biological medicines comes, however, at a considerable cost because of their generally high prices. The arrival of expensive innovative medicines increasingly challenges healthcare systems to find avenues to optimize spending while ensuring access to these therapies for their patients [1].

Following the expiry of exclusivity of a reference biological medicine, biosimilar alternatives can become available and introduce price competition in the market. As defined by the European Medicines Agency (EMA), a biosimilar is “a biological medicinal product that contains a version of the active substance of an already authorized original biological medicinal product (reference medicinal product)” [2]. Competition created by biosimilar entry has shown to result in decreased treatment costs and in some cases to facilitate patient access to biological therapies [3]. Moreover, savings derived from biosimilar competition may contribute to the financing of expensive innovative therapies [4].

In 2005, Europe took the lead in developing a tailored legal and regulatory pathway for the evaluation and approval of biosimilars [2, 5]. Fifteen years after the first biosimilar approval in Europe, over 60 biosimilars are approved for 16 distinct biological products across multiple therapeutic areas, including chronic inflammatory diseases and oncology [6].

Biosimilar development follows a different drug development paradigm compared to that of a new medicinal product. For a biosimilar, developers do not need to demonstrate de novo efficacy or safety, as these properties are well known and established for the reference product. Instead, biosimilars need to demonstrate high similarity in efficacy, safety and quality in relation to the reference product. Because of the inherent variability of biological medicines and the complexity of manufacturing, small differences may be present between a reference product and a biosimilar (which nota bene may also be the case between batches of the same biological). Biosimilars are thus highly similar but not identical versions of the reference product. In biosimilar development, it needs to be demonstrated that these small differences are not meaningful in terms of clinical outcomes. For this, regulators evaluate the totality of evidence gathered to demonstrate biosimilarity which finds its basis in an extensive physicochemical and biological characterization and comparison with the reference product. Biosimilar approval may rely in part on the knowledge of the reference product and is predominately based on comparative analytical and functional data, since this is a much more sensitive approach in detecting potential differences than a clinical study. As such, generally fewer clinical studies need to be carried out for a biosimilar than for the reference product [5, 7, 8].

After the evaluation and approval at European level, biosimilar market entry and implementation is organized by the individual European Member States. Biosimilar uptake varies among Member States, which may be partly explained by differing biosimilar market entry policies. In Belgium, 31 biosimilar products are reimbursed and available on the market [10], but biosimilar uptake is generally low compared to other European Union countries [3, 9–13].

As biosimilars represent a different development and approval paradigm, their acceptance by healthcare providers, patients and policy makers may require a change in mind-set [7]. Multiple studies have assessed the awareness and knowledge about biosimilars among healthcare providers and patients, identifying generally low to moderate levels of knowledge and trust in biosimilars and related concepts [14]. Whereas the knowledge and perceptions of healthcare providers and patients have been assessed in different regions of the world and European countries [14, 15], research with Belgian healthcare providers and patients is rather limited. Early Belgian policy oriented research identified a lack of awareness, a lack of information and concerns about interchangeability among healthcare providers as factors limiting biosimilar adoption in Belgium [16, 17] In 2017, the results of a survey among 41 Belgian rheumatologists revealed that they have doubts about the safety and efficacy of biosimilars and have concerns about their interchangeability with its reference product [18]. Interchangeable use refers to the possibility of exchanging one medicine for another medicine that is expected to have the same clinical effect. This could mean exchanging a reference product with a biosimilar (or vice versa) or replacing one biosimilar with another. When the prescriber decides to exchange, such practice is termed a “transition” or “switch”. If done at pharmacy level without consulting the prescriber, such an exchange is termed “(automatic) substitution” [5]. While switching biological reference products with their biosimilar or vice versa has become common practice, substitution of biologicals is largely not allowed or covered by a legal framework in Europe [19]. Also in Belgium, substitution of biologicals is not allowed [20].

Over previous years, several initiatives with the aim to increase biosimilar use have been implemented in Belgium. Policy actions include the establishment of a biosimilar usage target in hospitals, biosimilar use monitoring, and the stimulation of tender procedures. In 2018, a media campaign was launched to provide information on biosimilars to healthcare providers, patients and by extent the general public [12, 21–23]. This joint initiative by the Belgian competent authority and the reimbursement agency included the launch of a website with biosimilar information, patient leaflets and radio spots [22, 24]. Most of the above-mentioned measures focused on biosimilar use in hospitals, as the first available biosimilars are mainly used in the hospital in- or out-patient setting. Despite the multitude of policy initiatives taken, the use of biosimilars in Belgium continues to lag behind. Especially market shares of biosimilars dispensed in the community pharmacy are low [13, 25]. With the approval of biosimilars for high-value subcutaneously administered products (such as adalimumab and etanercept), biosimilars become increasingly available outside of the hospital setting in Belgium.

The arrival of biosimilars in ambulatory care comes with a distinct set of additional challenges compared to biosimilar use in the hospital context. The relative newness of biosimilars in this setting, the limited difference in list price between reference products and biosimilars, the lack of incentives for involved healthcare providers and patients to use biosimilars, possible differences in injection device, and the lack of an organized mechanism that may drive biosimilar use via tenders and facilitate switch management, as present in hospitals, may further complicate biosimilar use. Table 1 shows an overview of available biosimilars in ambulatory care in Belgium. An overview of relevant terminology used throughout this article is provided in Additional file 3.

**Table 1:** Biosimilars available in ambulatory care in Belgium (May 2021) [10]

INN	Product type	Net 2019 expenditure [26]	Reference product	Biosimilar	Reimbursement date biosimilar** [27]
Adalimumab*	TNF inhibitor	95.207.248	Humira®	Amgevita®	1/10/2018
				Hulio®	1/1/2019
				Hyrimoz®	1/1/2019
				Idacio®	1/10/2019
				Imraldi®	1/10/2018
Enoxaparin*	LMWH	22.446.229	Clexane®	Ghemaxan®	1/1/2021
Etanercept*	TNF inhibitor	45.197.777	Enbrel®	Benepali®	1/09/2016
				Erelzi®	1/7/2019
				Nepexto®	1/2/2021
Filgrastim	G-CSF	NPA	Neupogen®	Accofil®	1/6/2016
				Nivestim®	1/3/2014
				Tevagrastim®	1/2/2010
Follitropin alfa	FSH	NPA	Gonal-F®	Bemfola®	NA
				Ovaleap®	NA
Insulin glargine*	Long-acting insulin analogue	30.344.794	Lantus®	Abasaglar®	1/6/2016
Somatropin	Growth hormone	NPA	Genotropin®	Omnitrope®	1/4/2014

*G-CSF* granulocyte colony-stimulating factor, *INN* international non-proprietary name, *LMWH* low molecular weight heparin, *NPA* not publicly available, *NA* not applicable, non-reimbursed medicines, *FSH* follicle-stimulating hormone, *TNF* tumor necrosis factor

^*^Biologicals that are part of the top 25 medicine expenditures in the Belgian ambulatory sector. Insulin aspart is also part of this list (reference product: NovoRapid®, net 2019 expenditure: 22.229.748) and has an authorized biosimilar: Insulin aspart Sanofi®. Insulin aspart Sanofi® has, however, not (yet) been launched in Belgium [26]. Also the EU-approved biosimilars of teriparatide (reference product: Forsteo®, EU-approved biosimilars: Movymia®, Terrosa®, Livogiva®, Qutavina®) and insulin lispro (reference product: Humalog®, EU-approved biosimilar: Insulin Lispro Sanofi®) are not (yet) available on the market in Belgium [6, 26, 28]

^**^Reimbursement date of the first available product package

Considering that biosimilars are increasingly available for a new group of Belgian healthcare providers, especially community pharmacists and general practitioners, and the criticality of their role in the use of biosimilars, this study aims to investigate their knowledge and perception regarding biological medicines including biosimilars.

## Methods

### Survey design and data collection

Two sets of surveys, one for community pharmacists and one for physicians, were developed. The surveys were designed based on a review of the literature and consisted of five main parts: (i) participant characteristics, including experience with dispensing/prescribing biologicals in general and biosimilars in particular; (ii) knowledge about biosimilars; (iii) attitudes regarding dispensing/prescribing biologicals in general and biosimilars in particular; (iv) attitudes regarding interchangeability, switching, substitution, and (v) informational and educational needs. In the physician survey, a sixth category was included: (vi) attitudes regarding drivers and incentives for prescribing biosimilars. Questions were tailored to the particular stakeholder group. Participants received definitions on biological medicines, biosimilars, interchangeability, switching and substitution where appropriate. The surveys consisted predominately of closed multiple-choice questions. For some questions, multiple answers could be selected. The survey also included Likert scale questions, in which participants were asked to indicate their level of agreement with a proposed statement. The survey was tested in and adapted based on three pilot surveys. Both surveys were made available in Dutch and French to cater to the two main language regions in Belgium. The web-surveys were created using the online survey platform, SurveyMonkey®. The survey launched in November 2018 and closed in March 2019. Ethics approval was granted by the Research Ethics Committee UZ/KU Leuven (MP006667, Belgium).

### Participants

Two healthcare provider groups were targeted to participate: (i) community pharmacists and (ii) general practitioners and physician specialists who prescribe subcutaneous biologicals that are dispensed via the community pharmacy, for which an EMA evaluated and European Commission (EC) approved biosimilar alternative is available on the Belgian market (i.e., endocrinologists, rheumatologists, gastro-enterologists and dermatologists).

Healthcare providers across Belgium were invited to participate. Medical and pharmacy professional organizations on a national and regional level were asked to disseminate the survey among their members. The invitation to participate and the link to the online survey was subsequently included in newsletters and professional websites or social media pages of participating professional associations. In addition to this, community pharmacists involved in the training program of KU Leuven Master students Pharmaceutical Care received an invitation to participate. Additionally, participants were identified via the network of the research group.

### Data analysis

Results were analyzed descriptively for the overall participant group per stakeholder category. In the results section, relative numbers are presented as percentages and the considered sample size, which varied throughout the survey due to the logic applied in the survey questions and participant dropout, is included. Additional inferential statistics to test for differences between certain groups of interest (i.e., experienced and less experienced pharmacists and questions of interest (i.e., self-assessed competence in dispensing biologicals in general vs biosimilars in particular) were performed using Statistica software (Version 14). The Fisher exact test was used to compare proportions of categorical data. This test was chosen since the retrieved samples were small for certain questions. All tests were performed on a significance level of 5% (*α* = 0, 05), meaning *p*-values of lower than 0, 05 were considered significant.

## Results

To contextualize the results regarding the knowledge and perception of healthcare providers about prescribing or dispensing biosimilars, the surveys also enquired about their knowledge and perception regarding biological medicines in general.

### Community pharmacists

#### Participant characteristics

In total, 177 Belgian community pharmacists participated. All regions in Belgium were represented, although most participants worked in Flanders (86%, *n* = 153/177). Reponses were gathered across different age groups and most participants were female (69%, *n* = 123/177) (Table 2). Of the 177 participating community pharmacists, 115 completed the survey in full.

**Table 2:** Community pharmacists: participants’ characteristics

Characteristics	Community pharmacists		*n* =
	*n*	%	
Sex			177
Female	123	69	
Male	54	31	
Age			177
< 30 years	44	25	
> 30 years–45 years	56	32	
> 45 years–60 years	72	41	
> 60 years	5	3	
Years of experience as community pharmacist			177
< 2 years	18	10	
2–5 years	28	16	
6–10 years	23	13	
11–20 years	30	17	
21–30 years	59	33	
> 30 years	19	11	
Working region			177
Brussels	10	6	
Flanders	153	86	
Wallonia	14	8	
Working environment			177
*Multiple answers possible*			
Community pharmacy	177	100	
Professional pharmacist group	10	6	
University	1	1	
Other	3	2	

Percentages are rounded to the nearest integer

*N: *number

#### Experience with dispensing biologicals including biosimilars

Most pharmacists indicated to have experience with dispensing biological medicines (84%, *n* = 148/177). Not surprisingly, almost all had experience with dispensing Tumor Necrosis Factor (TNF)-alpha inhibitors (95%, *n* = 119/125) and hormones such as insulin, growth hormone, and follitropin-alpha (94%, *n* = 118/125), as both product classes are dispensed in the community pharmacy in Belgium (Additional file 2: Table S1).

A smaller portion of pharmacists had experience with dispensing biosimilars (32%, *n* = 45/139). Noteworthy, an identical number did not know whether they had yet dispensed a biosimilar or not. The majority who indicated to have dispensed a biosimilar, had experience with dispensing biosimilars of hormones (67%, *n* = 24/36) and TNF-alpha inhibitors (64%, *n* = 23/36) (Additional file 2: Table S2).

#### Knowledge about biosimilars

To evaluate the knowledge of pharmacists, respondents were asked about the accuracy of a few statements on biosimilar medicines (Additional file 1: Figure S1). The majority (67%, *n* = 95/142) correctly indicated a biosimilar to be highly similar in efficacy, safety, and quality to the reference product. Noteworthy, 18% (*n* = 25/142) had heard about biosimilars, but did not really know what the term means.

To test possible differences in knowledge about biosimilars between more recently graduated and more senior pharmacists, results of respondents with more versus less than 20 years of pharmacy experience were compared statistically (Additional file 2: Table S3). For none of the statements a statistically significant difference was found between less and more experienced pharmacists.

#### Attitudes about dispensing biologicals including biosimilars

**Fig. 1:**
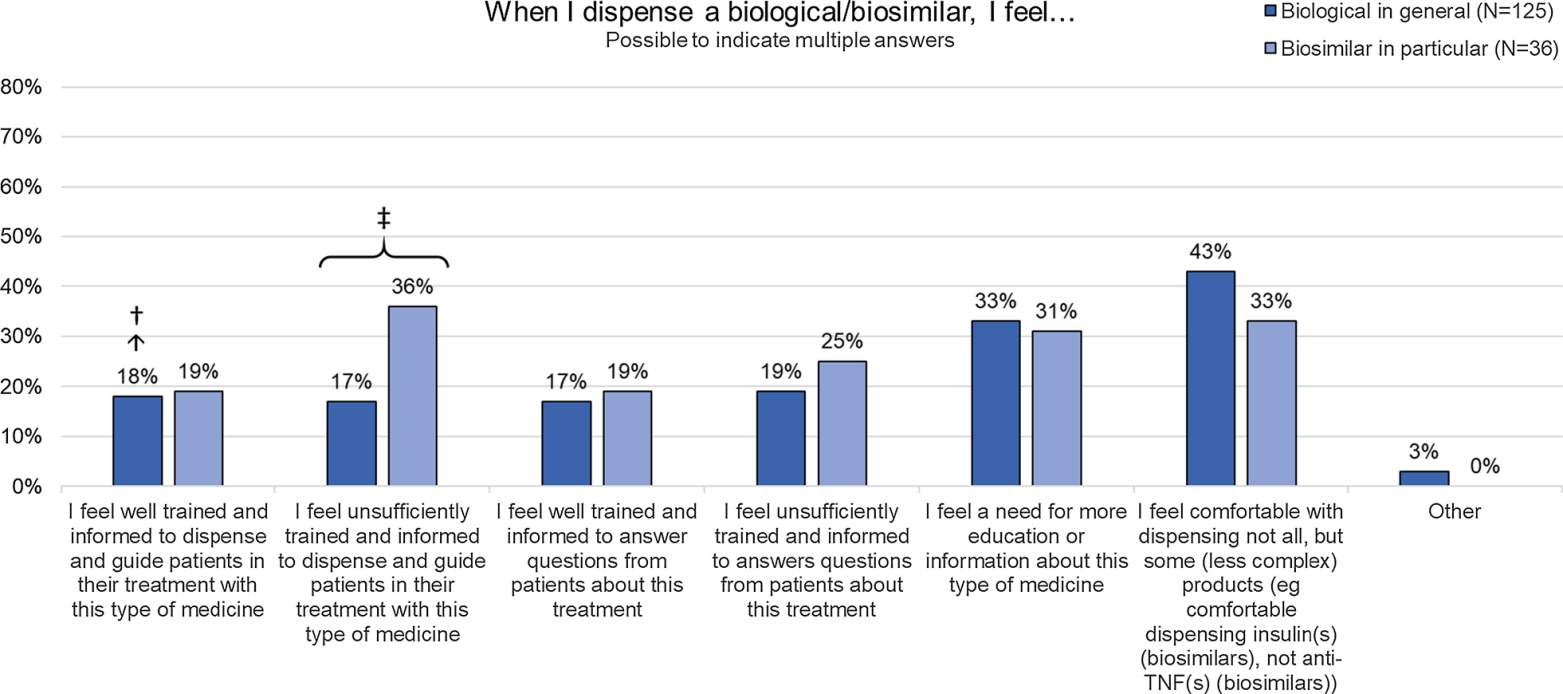
Self-assessed competence of community pharmacists to dispense biologicals (in general) and biosimilars (in particular). *Anti-TNF* anti-tumor necrosis factor, *N* number*.* Statistical testing: ‡: when testing for differences in self-assessed competence in dispensing biological medicines in general versus biosimilars in particular, a statistical difference was found for statement 2 (Additional file 2: Table S4). †: when testing for differences in self-assessed competences in dispensing biological medicines between more recently graduated and more senior community pharmacists (more (*N* = 47) versus less than 20 years (*N* = 78) of pharmacy experience), a statistical significant difference was found for statement 1 (Additional file 2: Table S5). When testing for differences in self-assessed competences in dispensing biosimilars between more recently graduated and more senior community pharmacists (more (*N* = 17) versus less than 20 years (*N* = 19) of pharmacy experience), no statistical significant difference were found for any of the statements (Additional file 2: Table S6)

When examining the self-assessed competency of pharmacists to dispense biologicals in general and biosimilars in particular (Fig. 1), over one-third felt only comfortable with dispensing less complex biologicals (43%, *n* = 54/125) and biosimilars (33%, *n* = 12/36), such as insulin but not the more complex anti-TNF products. About one-fourth of pharmacists (18%, *n* = 22/125) felt insufficiently trained to dispense and guide patients with their biological treatment. For biosimilars, this portion was larger (36%, *n* = 13/36). Pharmacists felt significantly less trained to dispense and guide patients with a biosimilar than a biological medicine in general (*p* = 0,023). For all other statements, no statistically significant difference in attitudes between dispensing biologicals in general and biosimilars in particular was found (Additional file 2: Table S4). Also, no significant differences were found in the self-assessed competency to dispense biologicals in general and biosimilars between pharmacists with more versus less than 20 years of experience as community pharmacist. Only when asked if they feel well trained and informed to dispense a biological, a statistically significant difference was found between groups (*p* = 0,032) (Additional file 2: Tables S5 and S6).

Only 22% (*n* = 23/103) agreed or fully agreed with the statement that they felt sufficiently trained to lead the dispensing discussion with the patient when dispensing biologicals (Additional file 1: Figure S2). For biosimilars in particular, this proportion was even lower (13%, *n* = 4/30). While the majority felt neutral about this statement, both for biologicals in general (46%, *n* = 47/103) and biosimilars (57%, *n* = 17), about 30% of pharmacists disagreed with the statement for both.

**Fig. 2:**
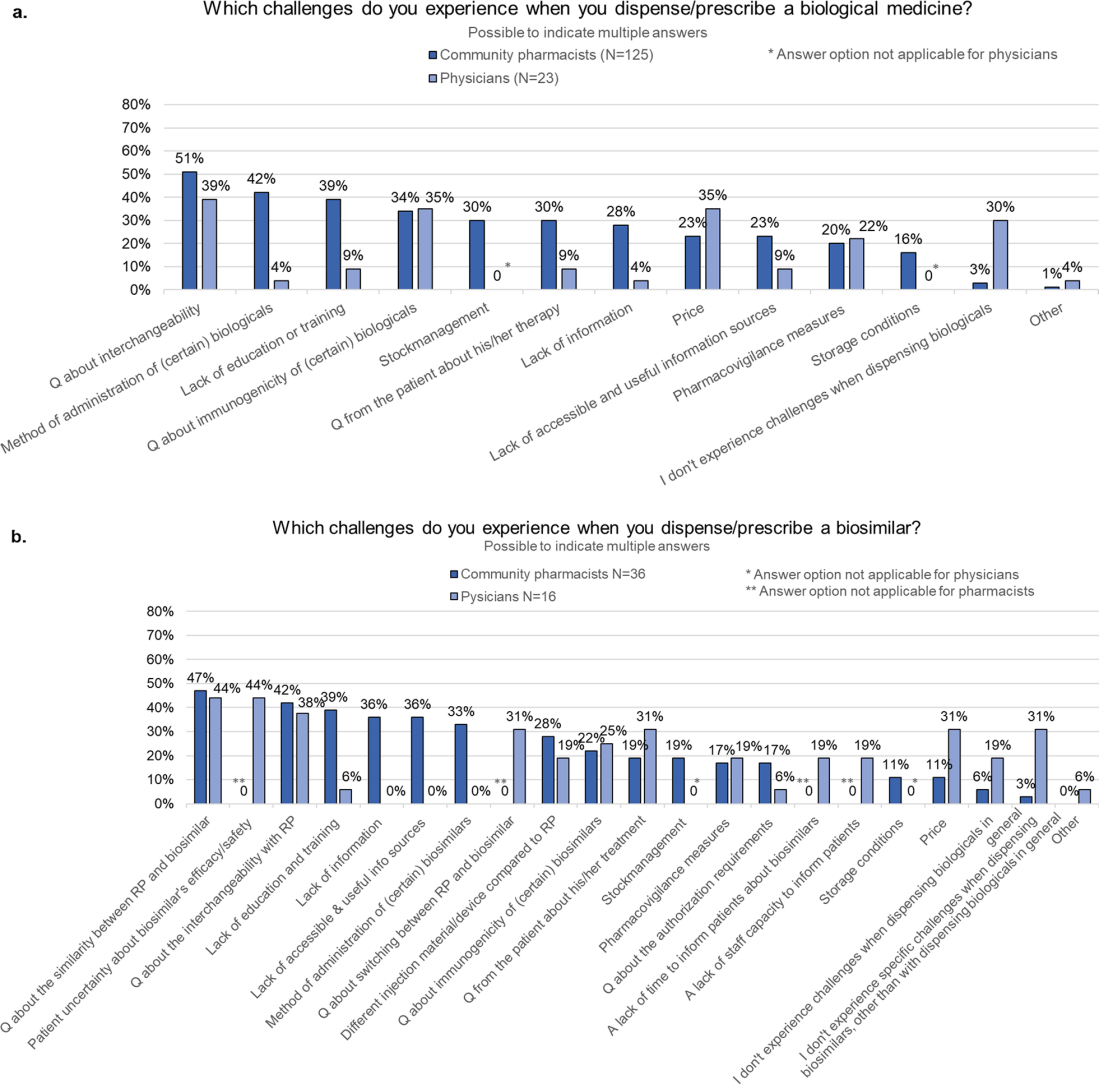
Perceived challenges when dispensing/prescribing a biological (in general) or a biosimilar (in particular) among community pharmacists and physicians. *N* number, *Q* questions,* RP* reference product

When asked which challenges they experience when dispensing a biological, pharmacists selected questions about interchangeability (51%, *n* = 64/125), the method of administration (42%, *n* = 53/125), lack of education and training (39%, *n* = 49/125), and immunogenicity (34%, *n* = 43/125). Only four pharmacists (3%) indicated to experience no challenges when dispensing biologicals (Fig. 2a).

With biosimilars, almost half of the pharmacists indicated to experience questions about the similarity of the biosimilar with its reference product (47%, *n* = 17/36) as challenging when dispensing. Over one-third also indicated to experience questions about interchangeability between the biosimilar and its reference product (42%, *n* = 15/36), a lack of education and training (39%, *n* = 14/36), a lack of accessible and useful information sources (36%, *n* = 13/36), and a general lack of information (36%, *n* = 13/36) here (Fig. 2b).

The majority of pharmacists (73%, *n* = 88/120) considered a counselling treatment conversation needed when a patient starts a treatment with a self-injectable biological. Approximately 50% considered it needed when a patient switches from a self-injectable reference biological product to a biosimilar or vice versa (Additional file 1: Figure S3).

#### Attitudes about interchangeability, switching, and substitution

**Fig. 3:**
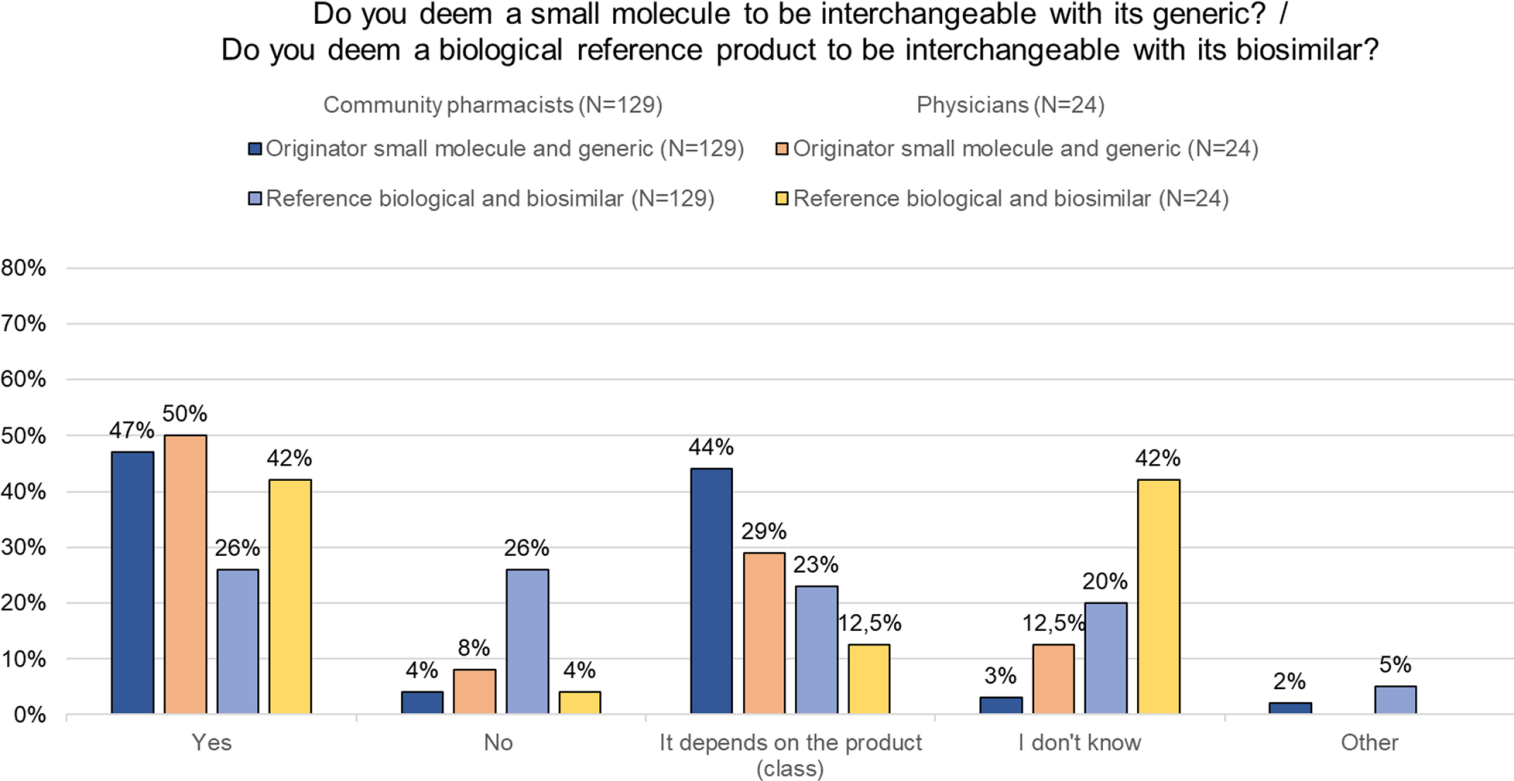
Community pharmacist and physician views on interchangeability. Interchangeability: interchangeability refers to the possibility of exchanging one medicine for another medicine that is expected to have the same clinical effect. This could mean replacing a reference product with a biosimilar (or vice versa) or replacing one biosimilar with another. *N* number, *RP* reference product

Approximately half of pharmacists (47%, *n* = 60/129) deemed a small molecule medicine to be interchangeable with its generic (Fig. 3). For reference biological and biosimilar medicines this percentage dropped to about one quarter (26%, *n* = 33/129).

About one-third of pharmacists (35%, *n* = 45/129) believed that an authorized biosimilar can be considered interchangeable with its reference product. The majority (60%, *n* = 77/129) believed that additional data are needed to demonstrate interchangeability besides these for marketing authorization (Additional file 1: Figure S4a). Opinions about the interchangeability of two biosimilars of the same reference product differed (Additional file 1: Figure S4b).

**Fig. 4:**
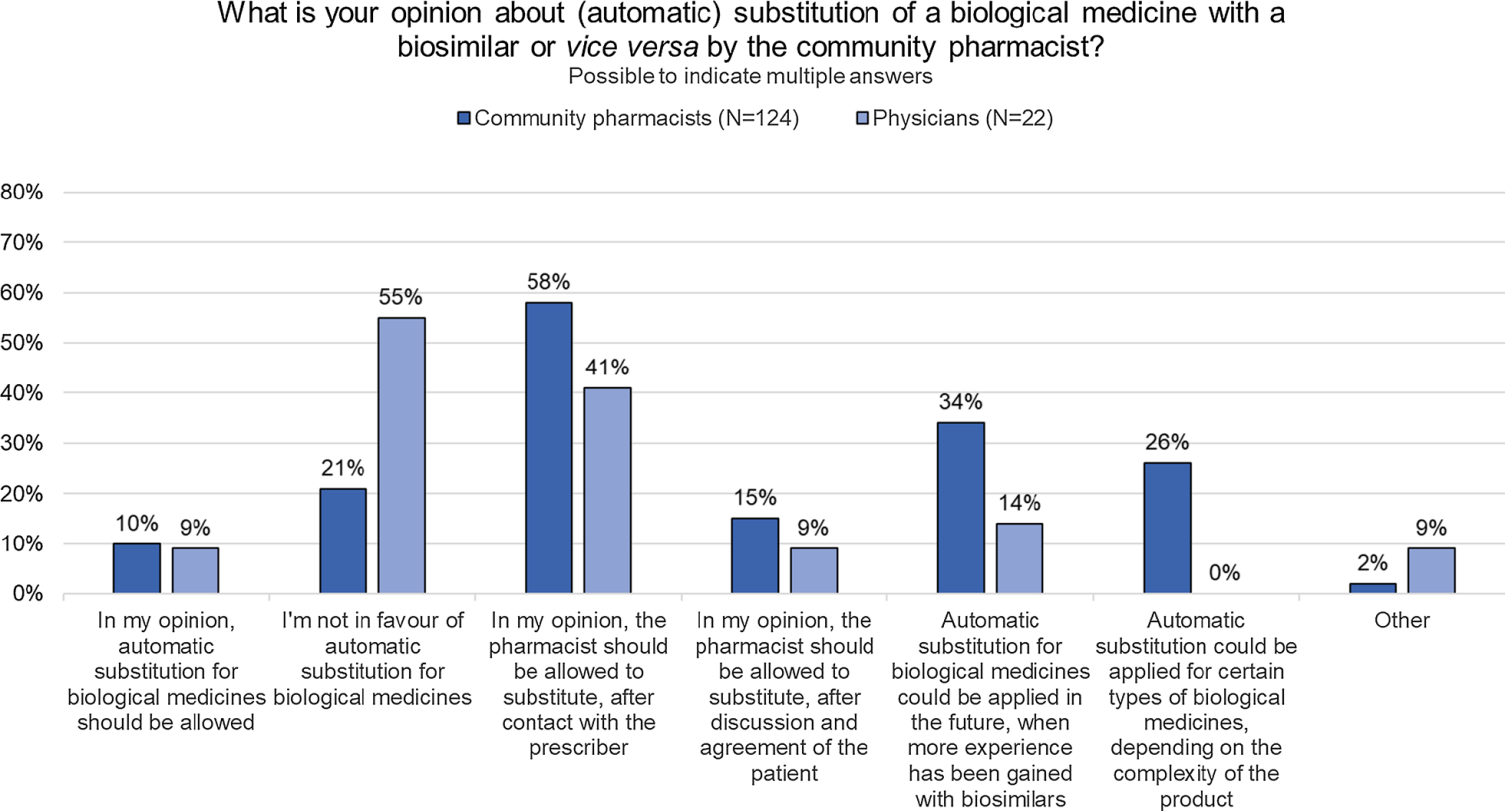
Questions about (automatic) substitution to community pharmacists and physicians. Automatic substitution: the pharmacist dispenses one medicine instead of another equivalent and interchangeable medicine at pharmacy level without consulting the prescriber. *N* number, *RP* reference product

Over half of pharmacists (58%, *n* = 72/124) indicated to believe that they should be allowed to substitute the original biological with its biosimilar, after contacting the prescribing physician (Fig. 4). Over a third (34%, *n* = 42/124) believed that automatic substitution could be applied in the future when more experience has been gained with biosimilars, while 26% (*n* = 32/124) thinks automatic substitution can be applied, depending on the complexity of the product. Only 10% (*n* = 13/124) believed that substitution between biological reference and biosimilar medicines should be allowed automatically.

When asked to what extent they agreed with the statement that substitution of biological medicines could be done, after contact with the prescriber, the majority (73%, *n* = 91/124) either agreed or fully agreed (Additional file 1: Figure S5a). Switching from a biological reference product to a biosimilar or vice versa should remain the responsibility of the prescribing physician, according to 66% (*n* = 82/124 agreed or fully agreed with the statement) of pharmacists.

#### Informational and educational needs

Less than half of pharmacists (42%, *n* = 47/113) had followed a training or educational symposium about biologicals in the past. A smaller portion had followed one specifically about biosimilars (22%, *n* = 25/113) (Additional file 2: Tables S1 and S2).

**Fig. 5:**
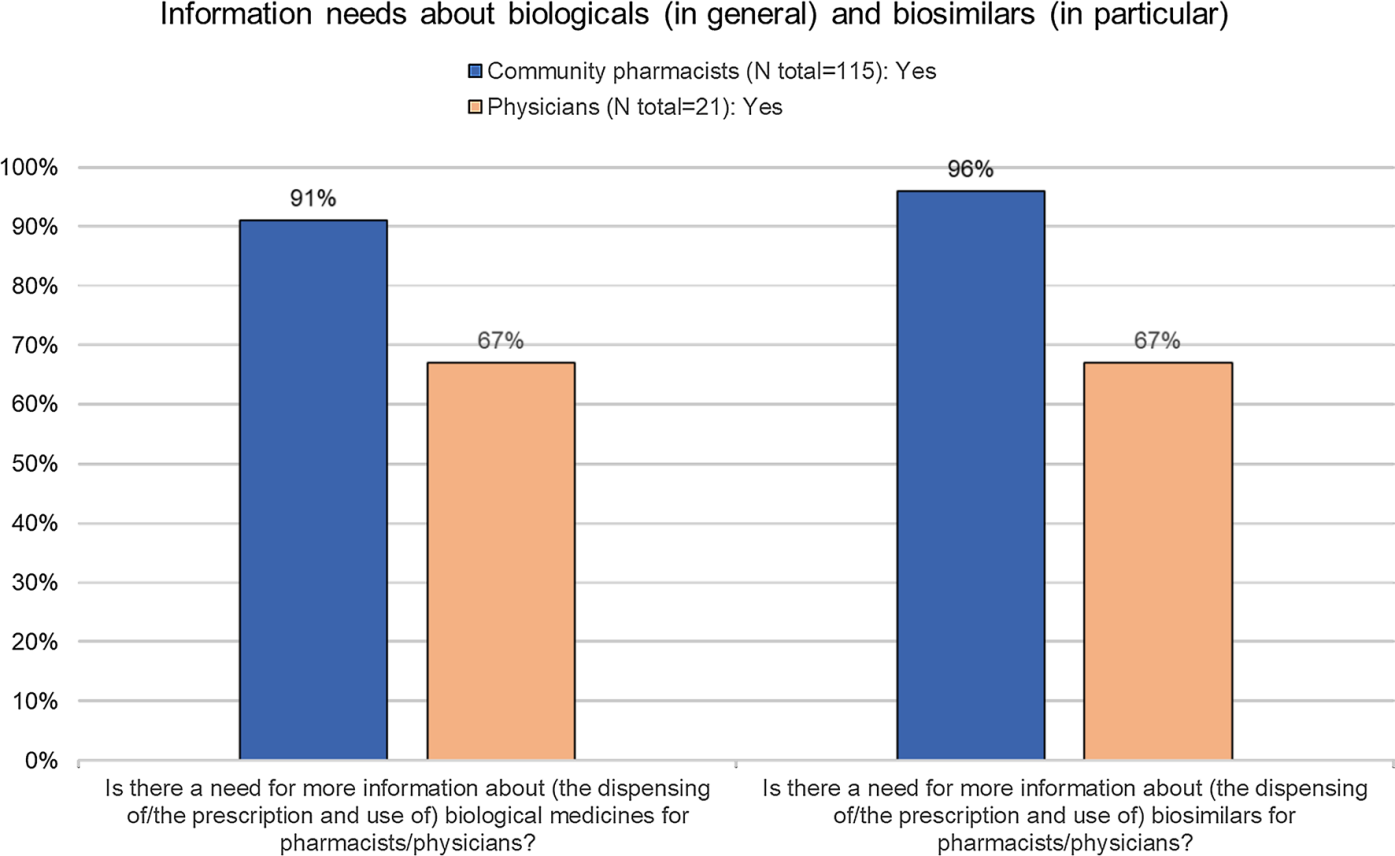
Information need about biologicals in general and biosimilars in specific among community pharmacists*. EMA* European Medicines Agency, *FAMHP* Federal Agency for Medicines and Health Products (Belgian National Competent Authority),* KOL* key opinion leader,* LMWH* low molecular weight heparins, *N* number,* NIHDI* National Institute for Health and Disability Insurance (Belgian national health insurer), *TNF-alfa blockers* tumor necrosis factor-alfa blockers

A high need for information about biologicals in general (91%, *n* = 105/115), and biosimilars in particular (96%, *n* = 110/115) was expressed (Fig. 5). Almost all (95%, *n* = 107/115) indicated they would like to follow a training about biological including biosimilar medicines (Additional file 1: Figure S6a). Pharmacists expressed interest in almost any type of the suggested topics (Additional file 1: Figure S6b). When asked by which organization they prefer to be informed, national or regional professional organizations were ranked first by more than half (51%, *n* = 59/115) (Additional file 1: Figure S6c).

### Physicians

#### Participant characteristics

In total, 30 physicians participated in this study. Most worked in Flanders (80%, *n* = 24/30), while a minority worked in Wallonia (20%, *n* = 6/30). Physicians were specialized in a variety of therapeutic domains, including rheumatology (33%, *n* = 10/30), dermatology (17%, *n* = 5/30) and general practice (13%, *n* = 4/30) (Table 3).

**Table 3:** Physicians: participants’ characteristics

Characteristics	Physicians		*n* =
	*n*	%	
Sex			30
Female	17	57	
Male	13	43	
Age			30
< 30 years	3	10	
> 30 years–45 years	14	47	
> 45 years–60 years	9	30	
> 60 years	4	13	
Years of experience as physician			30
0–5 years	4	13	
6–10 years	4	13	
11–20 years	11	37	
21–30 years	5	17	
> 30 years	6	20	
What is your function?			30
*Multiple answers possible*			
Rheumatologist	10	30	
Dermatologist	5	17	
Gastro-enterologist	2	7	
Endocrinologist	1	3	
General practicioner	4	13	
Representative medical association	1	3	
Other	7	23	
Working region			30
Brussels	0	0	
Flanders	24	80	
Wallonia	6	20	
Working environment			30
*Multiple answers possible*			
Private practice	12	40	
University hospital	5	17	
General hospital	15	50	
Professional medical association	1	3	
University	1	3	
Other	1	3	

Percentages are rounded to the nearest integer

*N: *number

#### Experience with prescribing biologicals including biosimilars

The majority of physicians (77%, *n* = 23/30) had experience with prescribing biological medicines. A few (10%, *n* = 3/30) did not know if they had prescribed a biological medicine (Additional file 2: Table S7).

Over half of physicians (52%, *n* = 14/27) had prescribed a biosimilar. Seven percent indicated to have not prescribed a biosimilar, but to follow a patient under treatment with a biosimilar (*n* = 2/27). The majority (67%, *n* = 16/24) did not have experience with switching a patient under treatment with a reference product to a biosimilar (Additional file 2: Table S8).

#### Knowledge about biosimilars

Most physicians recognized that biosimilars are similar in efficacy, safety, and quality with respect to their reference medicine (63%, *n* = 17/27). About ten percent had heard about biosimilars, but did not know what the term exactly means (11%, *n* = 3/27) (Additional file 1: Figure S1).

#### Attitudes about prescribing biologicals including biosimilars

When asked about challenges that they experience when prescribing biologicals in general, most physicians indicated to have questions about the interchangeability of biological medicines (39%, *n* = 9/23). The price of biological medicines (35%, *n* = 8/23) and questions about their immunogenicity (35%, *n* = 8/23) were also recognized as challenging (Fig. 2a).

When prescribing biosimilars, physicians indicated to experience questions about the similarity of biosimilars with their reference product (44%, *n* = 7/16), uncertainties of patients about the efficacy and safety of biosimilars (44%, *n* = 7/16), and questions about interchangeability between biosimilars and their reference product (38%, *n* = 6/16) (Fig. 2b) as challenges.

#### Attitudes about interchangeability, switching, and substitution

Half of physicians believed generic medicines and their original product are interchangeable (50%, *n* = 12/24). This proportion was smaller for biosimilar and reference products (42%, *n* = 10/24) (Fig. 3). The majority was of the opinion that additional data are needed to demonstrate that a biosimilar is interchangeable with its reference product upon authorization (63%, *n* = 15/24) (Additional file 1: Figure S4a).

Most physicians were not in favor of substitution in an automatic way (55%, *n* = 12/22). About 40% believed that the pharmacist should be allowed to substitute after contact with the prescriber (41%, *n* = 9/22) (Fig. 4).

The majority of physicians (77%) disagreed or fully disagreed with the statement that substitution of a biological reference product with a biosimilar could be done by the general practitioner after contact with the initiating prescriber. Surprisingly, the percentage of physicians disagreeing was lower (45%, *n* = 10/22) when asked if they agreed this could be done by the pharmacist, after contact with the prescriber. Regarding switching, 36% of physicians disagreed or fully disagreed with the statement that insufficient data are available about switching between biological reference products and biosimilars (*n* = 8/22), while 32% was neutral and 32% agreed with the statement (*n* = 7/22). (Additional file 1: Figure S5b).

#### Informational and educational needs

About 70% (*n* = 15/21) of physicians indicated to have followed a training or symposium about biologicals in general, and over half (52%, *n* = 11/21) specifically about biosimilars. (Additional file 2: Tables S7 and S8).

The majority indicated a need for more information both about (the prescription and use of) biologicals in general and biosimilars in particular (67%, *n* = 14/21) (Fig. 5). When asked by which organization they would like to be informed, the national or regional professional physician association was ranked first, followed by a European professional physician association (Additional file 1: Figure S6d). When asked which organization would be suited to draft guidance about biosimilar use, also the national or regional professional physician association was ranked first. (Additional file 1: Figure S6e).

#### Attitudes about drivers and incentives for prescribing biosimilars

**Fig. 6:**
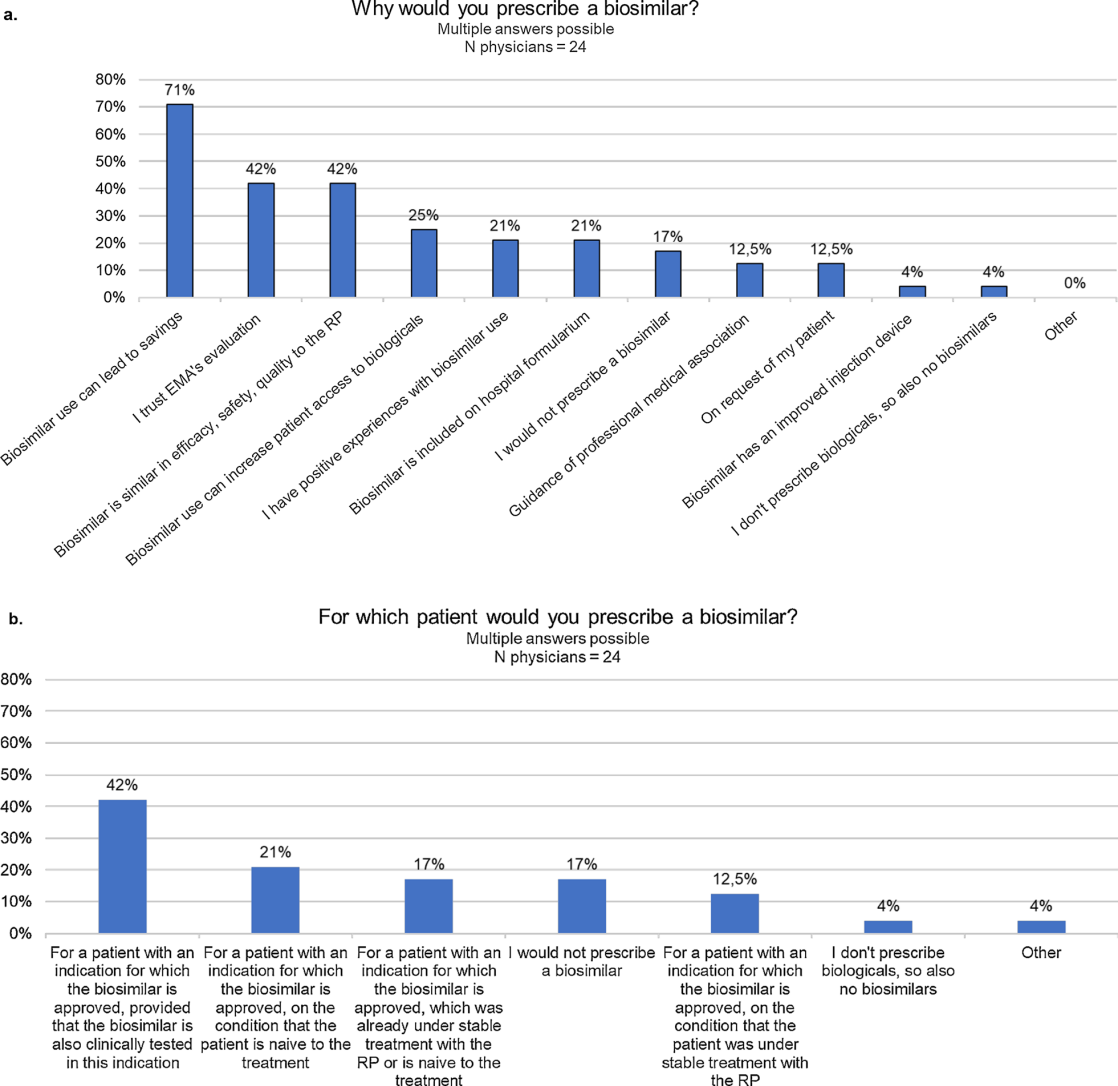
Questions about the motivation of physicians to prescribe biosimilars. *EMA* European Medicines Agency,* N* number, *RP* reference product

When asked for which reasons they would prescribe a biosimilar, the majority of physicians mentioned savings as reason (71%, *n* = 17/24). Confidence in the evaluation of biosimilars by the EMA (42%, *n* = 10/24), the fact that the biosimilar is similar compared to the reference product (42%, *n* = 10/24) and a potential increase in patient access to biological therapies (25%, *n* = 6/24) were also selected (Fig. 6a). When asked for which patient they would prescribe a biosimilar, 42% (*n* = 10/24) would only prescribe the biosimilar if it was clinically tested for the specific indication of their patient or only for bio-naïve patients (21%, *n* = 5/24) (Fig. 6b). When asked about reasons not to prescribe biosimilars, 42% (*n* = 10/24) selected the argument that the product is less clinically tested compared to the reference product, uncertainty of their patient (33%, *n* = 8/24), and a lack of knowledge about the biosimilar concept and its evaluation (29%, *n* = 7/24) (Additional file 1: Figure S7).

The majority of physicians recognized the need for some kind of incentive (55%, *n* = 12/22) to stimulate biosimilar prescription in the ambulatory setting (Additional file 1: Figure S8a). When asked what kind of incentive is expected, information about biosimilars by regulatory authorities, transparency about the realized savings derived from biosimilar market entry, and additional budget for staff to support biosimilar implementation were most frequently indicated (Additional file 1: Figure S8b). The majority (55%, *n* = 12/22) believed that a similar initiative like the Convenant “Restart biosimilar medicines in Belgium”, which aimed to stimulate the use of biosimilars in the hospital, would be useful to stimulate the prescription of biosimilars in the ambulatory setting (Additional file 1: Figure S8c).

## Discussion

Since biosimilar use has been predominately a hospital matter in previous years, earlier research in the domain of stakeholder knowledge about biosimilar medicines generally focussed on the knowledge and perception of hospital pharmacists and physician specialists [14]. With the approval of biosimilars for subcutaneously administered biologicals (such as adalimumab, etanercept and insulin), biosimilars are finding their way to the community pharmacy in Belgium.

In view of the loss of exclusivities of different biologicals with high (therapeutic) value in the ambulatory care setting and the subsequent emergence of biosimilars, this study quantitatively assessed the knowledge and perceptions of Belgian community pharmacists and physicians about biological including biosimilar medicines in this particular setting.

### A clear need for active educational and informational measures

The level of knowledge and understanding about biosimilars among Belgian community pharmacists was noted to be low. Also for physicians, a need for educational initiatives was clearly expressed. The results of this study show that there is a substantial demand for more information and education about different aspects about biological including biosimilar medicines for this group of Belgian healthcare providers.

These findings are consistent with previous research on healthcare provider perceptions about biosimilars, showing low to moderate knowledge and trust towards biosimilars across varying specialisms and countries [14, 15]. While earlier research largely investigated the perspective of healthcare professionals active in the hospital context, a French web-based survey included also the perspective of community pharmacists in their study. Their 2017 survey results showed that about half of community pharmacist survey participants were “not at all” informed about biosimilars, compared to less than one fifth of participating hospital pharmacists [29]. Even though experience with biosimilars may have grown in the ambulatory care setting over the past few years, the results of this current Belgian websurvey indicate that the self-assessed knowledge among community pharmacists about biosimilars is still limited in Belgium.

A survey conducted among Irish general practitioners in 2017 showed that 60% of participants were unable to define a biosimilar or had never heard of the term [30]. A survey among Belgian rheumatologists in 2016, pointed at information gaps and doubts about biosimilar medicines. In particular, concerns about the interchangeability of biological reference products with biosimilars were found [18]. A general lack of familiarity and trust in biosimilar medicines among Belgian physicians was already observed in 2013 [16, 17]. The findings of this study show that physicians’ uncertainty about biosimilars may have not been sufficiently addressed over the past few years.

Moreover, and more surprisingly, this study indicates that community pharmacists and physicians not only face challenges with dispensing or prescribing biosimilars but also with biological medicines in general. This statement is supported by the statistical analysis, where nearly no significant differences were found between the self-assessed competences between dispensing biological medicines in general and biosimilar medicines in particular among community pharmacists.

It is essential that physicians and community pharmacists are well trained to make treatment decisions and counsel patients regarding biological, including biosimilar, therapies. Although the biosimilar drug development and regulatory assessment paradigm originates from 2005 and the first biosimilar approval dates from 15 years ago, physicians still ask for clinical studies and pharmacists have questions about similarity and interchangeability concepts. As biosimilars are relatively recently available in ambulatory care in Belgium and their market shares remain low, general practitioners and community pharmacists may have been only confronted to a limited extent with biosimilars in clinical practice. Misunderstandings about biosimilar concepts may also stem from a lack of knowledge about biological medicines in general [14]. Nonetheless, physicians and community pharmacists have the responsibility to prescribe/dispense these medicines in an informed and knowledgeable manner, and adequately counsel patients with their treatment. Healthcare providers are expected to keep up with developments in pharmaceutical therapies that enter clinical practice. University curricula should prepare physicians and pharmacists with up-to-date education and continuous education should provide support with lifelong learning during their professional career.

The results of this study ask for an examination of the existing education and outreach on biological medicines and biosimilars for Belgian healthcare providers. Whereas earlier research suggested that information lacks [17], informational material on biosimilars is now abundantly available. This suggest that the available informational material does not effectively reach the physician and pharmacist [14].

An important responsibility lies with the Belgian professional associations to disseminate objective information about biosimilars, and include biosimilar medicines in the continuing education of physicians and pharmacists. The emphasis should be on organizing (mandatory) educational sessions in the framework of lifelong learning, rather than making information passively available. Professional associations and policy makers should collaborate to facilitate a coherent stream of information and develop targeted educational measures to reach healthcare providers maximally. This may benefit from a pro-active and centrally coordinated approach from the Belgian national medicines agency and the Ministry of Social Affairs and Health.

In addition to the importance of permanent education courses to continuously update knowledge and insights of healthcare providers on emerging and evolving topics, the university curricula for future healthcare providers warrant a closer look. A follow-up study investigated the knowledge of Belgian medicine and pharmacy students about biologicals including biosimilars. Only low to moderate percentages of master students (ranging from 2 to 42%) appear to feel well prepared to work with biologicals in general and biosimilars in particular in the future [31, 32]. Compared to master students Medicine and Pharmaceutical Care, Master students Drug Development seem to be more informed [31, 32]. This survey also showed no statistical difference in terms of knowledge about biosimilars between more recently graduated and more senior community pharmacists. Based on an examination of the presence of biological including biosimilar topics in the learning objectives of the pharmacists’ curriculum, it should be considered to expand training on this [31, 32]. University education committees should appraise the courses within the Master’s degrees regarding biological including biosimilar medicines and expand and update content where needed to prepare future healthcare providers with the necessary knowledge and competencies to prescribe/dispense these medicines [31].

### Substitution of biological medicines

Whereas the marketing authorization of biosimilars is based on the recommendation of the EMA and the decision of the EC, decisions on interchangeability and substitution are made at the Member State level [5]. Similarly to most European countries, pharmacy-level substitution is not allowed for biological medicines in Belgium [20]. In this study, 58% of community pharmacists indicated to be in favor of introducing substitution for biological medicines, albeit after contacting the prescriber. Also 41% of physicians seemed to be in favor of substitution by the community pharmacist, if done with the prescriber’s approval.

Substitution could be a potential strategy to stimulate biosimilar usage in ambulatory care. Since biosimilars have proven to be equally effective and safe as their reference product when they enter the market, substitution has become an organizational or political challenge rather than a scientific one [33]. For example, in France and the US, pharmacist-led substitution for biologicals is legislatively possible [15, 19, 34]. Some other European countries have new legislation planned to allow pharmacist-led substitution for (certain) biologicals [19, 35]. Before this could be explored in the Belgian context, the demand for more information about biological including biosimilar medicines should be met to ensure that involved healthcare providers are well trained to counsel patients regarding biosimilar use and manage such an exchange. An earlier study among Finnish healthcare providers has pointed out several issues, and ways to solve them, regarding the implementation of substitution for biological medicines [34]. Similar to the results of this study, specific educational requirements for all stakeholders involved in the substitution process were underlined as a condition [34]. Community pharmacists and pharmacy staff should be educated and trained to counsel patients including device training. Substitution may also facilitate stock management as it limits the number of expensive biological medicines that must be stocked in the community pharmacy [34].

### The role and design of healthcare provider incentives

Besides the knowledge of healthcare providers, other factors may influence the adoption of biosimilars in clinical practice. Next to healthcare providers’ uncertainty and questions regarding biosimilars, low biosimilar use may be explained by the fact that physicians identify no or insufficient benefits to prescribe biosimilars and change their patients in the ambulatory context. Whereas in the hospital setting the use of biosimilars is determined to a large extent by tender mechanisms, no such driver exists in the ambulatory care setting. As the difference in the list price between originator biologicals and biosimilars is generally limited in Belgium [10], physicians may not recognize direct benefits from prescribing a biosimilar. As a considerable proportion of patients treated with biosimilars in the Belgian ambulatory setting are initiated in the physician’s private practice, incentive schemes outside of hospital-level incentives may be required.

The majority of physicians in this survey confirmed the need for prescriber incentives to support biosimilar usage in the ambulatory context. This finding is consistent with previous papers also pointing out the need for tangible incentives for healthcare providers [33, 36]. Following the emergence of biosimilars in the ambulatory care setting, a pilot financial incentive was introduced in 2019 by the Belgian national health insurer linked to the prescription of etanercept and adalimumab biosimilars [37]. The incentive has been discontinued because of its limited success [37]. The current study reveals that prescriber incentives should not necessarily be monetary in nature, as participants ranked informational and educational support as first preferred incentive. When implementing an incentive, it should aim to improve patient care rather than to provide a financial benefit at the level of the individual physician [33, 36]. Budget to remunerate additional staff to support the implementation of biosimilars could serve as a tangible method to incentivize prescribers.

In France such a gain sharing incentive was launched in 2018, as part of their national strategy aiming to achieve 80% biosimilar uptake by 2022 [38]. For biologicals such as adalimumab and etanercept, the initiation of treatment in France is done at the hospital, after which the initiated product is continued in the ambulatory setting [38]. The initiation in the hospital thus influences the subsequent use in the ambulatory setting. Therefore this incentive targeted the hospitals by rewarding them with 20% of the price difference between the originator biological and biosimilar for every insulin glargine, etanercept and adalimumab biosimilar prescribed in the hospital and dispensed in the community pharmacy or for every renewed prescription in the ambulatory setting resulting from the initiation in the hospital [38]. Preliminary results showed a positive effect on biosimilar market shares [39, 40]. Similarly in Ireland, the introduction of a prescribing incentive in the form of a gain-share of €500 per patient initiated or switched to a best-value adalimimab and etanercept was reported to have contributed significantly to an increase in biosimilar use [41].

In addition to a gain sharing incentive, the results presented here indicate that efforts should be made to report transparently about the generated savings from biosimilar competition and how they are used.

### Study strengths and limitations

This study was the first to examine the level of knowledge and perception of Belgian community pharmacists about biologicals including biosimilars. The relatively large sample size (a sample size of 177 participants allows to report for the population of 9200 Belgian community pharmacists with a confidence level of 95% and error margin of 7.3%), representative distribution of the sex (70% female in both the general Belgian community pharmacist population and survey sample) and fair distribution of age groups among participating pharmacists ensure that these results are indicative for the larger population of Belgian community pharmacists [42–44].

Limitations to the survey include the fact that mainly pharmacists and physicians working in Flanders participated, and the limited sample of participating physicians. Because participants were mainly recruited via professional organizations, a response rate could not be calculated. In addition, one could argue that pharmacists and physicians that showed interest—and participated—in this survey may have a higher level of knowledge about biosimilars than the overall healthcare provider population in Belgium. Moreover, earlier research showed that the flow of information and the knowledge about biosimilars may be higher among Flemish physicians compared with Wallonian physicians [45]. The results of this survey might therefore be even an overestimation of the actual level of awareness on biosimilars, which was already reported to be low in this study.

### Future perspectives

Initiatives and incentives targeted at pharmacists and physicians should play a central role in future policy making to support biosimilar usage in ambulatory care. As drivers appear to be lacking for biosimilar use in ambulatory care in Belgium, the exploration of policies and incentives in the ambulatory care context is becoming more pertinent. A continuing low use of biosimilars may deter companies to launch future biosimilars on the Belgian market. In Belgium, no insulin lispro biosimilar has been launched so far. Also, Mylan’s insulin glargine biosimilar (Semglee®) is not marketed in Belgium [10]. Belgian policymakers should work closely together with healthcare providers to create incentives tailored to their needs, in order to create a balanced climate for off-patent biological and biosimilar medicines.

In addition to policy actions, the university curricula should be fit for purpose to prepare physicians and pharmacists for their prescribing and dispensing responsibility of best-value biologicals (both reference biologicals and biosimilars). Education should include elements on cost-effective medicine use.

Besides the perspective of Belgian healthcare providers, the views of Belgian patients should be assessed. The perspectives of ambulatory care patients are currently being investigated in a subsequent study commissioned by the Belgian national health insurer [46]. The results of these two studies can inform the development of new educational initiatives to stimulate biosimilar adoption in clinical practice, tailored to the needs of both Belgian healthcare providers and patients.

## Conclusions

This study shows that Belgian community pharmacists and physicians have considerable uncertainties with prescribing and dispensing biological medicines in general and biosimilars in particular. It appears that healthcare provider knowledge gaps about biosimilars have not been sufficiently addressed over previous years. Targeted educational measures that actively reach Belgian community pharmacists and physicians are required to reduce the information gap. Equally, policy interventions to stimulate the use of biosimilar medicines will be needed to ensure that Belgium captures their societal benefits over the longer term. The results of this study can inform the design of necessary educational and policy measures to support biosimilar use in ambulatory care in Belgium.

## Supplementary Information

**Additional file 1: Figure S1.** Familiarity with and knowledge about biosimilars among community pharmacists and physicians. **Figure S2.** Additional question on the self-assessed competence of community pharmacists to dispense biologicals (in general) and biosimilars (in particular). **Figure S3.** Opinion of community pharmacists about the organisation of a counselling treatment conversation for patient biosimilar use. **Figure S4.** Additional questions posed to community pharmacists and physicians regarding interchangeability. **Figure S5.** Additional questions posed to community pharmacists and physicians regarding substitution. **Figure S6.** Additional questions posed to community pharmacists and physicians regarding information and training needs. **Figure S7.** Reasons why physicians would not prescribe a biosimilar. **Figure S8.** Questions about the need for incentives to stimulate biosimilar prescription in the ambulatory setting.

**Additional file 2: Table S1.** Community pharmacists: participants’ experience with biologicals in general. **Table S2**. Community pharmacists: participants’ experience with biosimilars. **Table S3**. Statistical analysis—testing for differences between experienced and more recently graduated pharmacists in terms of knowledge about biosimilars. **Table S4.** Statistical analysis—testing for differences between the self-assessed competences of pharmacists in dispensing biologicals in general versus biosimilars in particular. **Table S5**. Statistical analysis—testing for differences between the self-assessed competences in dispensing biologicals in general between experienced and more recently graduated pharmacists. **Table S6.** Statistical analysis—testing for differences between the self-assessed competences in dispensing biosimilars between experienced and more recently graduated pharmacists. **Table S7.** Physicians: participants’ experience with biologicals in general. **Table S8.** Physicians: participants’ experience with biosimilars.

**Additional file 3:** **Supplementary Box 1** Key terminology.

## Data Availability

All data generated or analyzed during this study are included in this published article and its supplementary information files.

## Abbreviations

EMA: European Medicines Agency
EC: European Commission
TNF: Tumor necrosis factor
