# Supplementary material for: Knowledge and perception of biosimilars in ambulatory care: a survey among Belgian community pharmacists and physicians
Source: J Pharm Policy Pract. 2021 Jun 22;14:53. doi: 10.1186/s40545-021-00330-x (PMC8218462; doi:10.1186/s40545-021-00330-x)
Supplement: Supplementary file 1 — Additional file 1: Figure S1. Familiarity with and knowledge about biosimilars among community pharmacists and physicians. Figure S2. Additional question on the self-assessed competence of community pharmacists to dispense biologicals (in general) and biosimilars (in particular). Figure S3. Opinion of community pharmacists about the organisation of a counselling treatment conversation for patient biosimilar use. Figure S4. Additional questions posed to community pharmacists and physicians regarding interchangeability. Figure S5. Additional questions posed to community pharmacists and physicians regarding substitution. Figure S6. Additional questions posed to community pharmacists and physicians regarding information and training needs. Figure S7. Reasons why physicians would not prescribe a biosimilar. Figure S8. Questions about the need for incentives to stimulate biosimilar prescription in the ambulatory setting. [file 40545_2021_330_MOESM1_ESM.docx]

**Knowledge and perception of biosimilars in ambulatory care: A survey among Belgian community pharmacists and physicians**

Liese Barbier, Yannick Vandenplas, Steven Simoens, Paul Declerck, Arnold G. Vulto, Isabelle Huys

Journal of Pharmaceutical Policy and Practice

Contact: liese.barbier@kuleuven.be

**Supplementary Figures**

- **Figure S1** Familiarity with and knowledge about biosimilars among community pharmacists and physicians
- **Figure S2** Additional question on the self-assessed competence of community pharmacists to dispense biologicals (in general) and biosimilars (in particular)
- **Figure S3** Opinion of community pharmacists about the organisation of a counselling treatment conversation for patient biosimilar use
- **Figure S4** Additional questions posed to community pharmacists and physicians regarding interchangeability
- **Figure S5** Additional questions posed to community pharmacists and physicians regarding substitution
- **Figure S6** Additional questions posed to community pharmacists and physicians regarding training needs
- **Figure S7** Questions about the need for incentives to stimulate biosimilar prescription in the ambulatory setting

**Figure S1.** Familiarity with and knowledge about biosimilars among community pharmacists and physicians


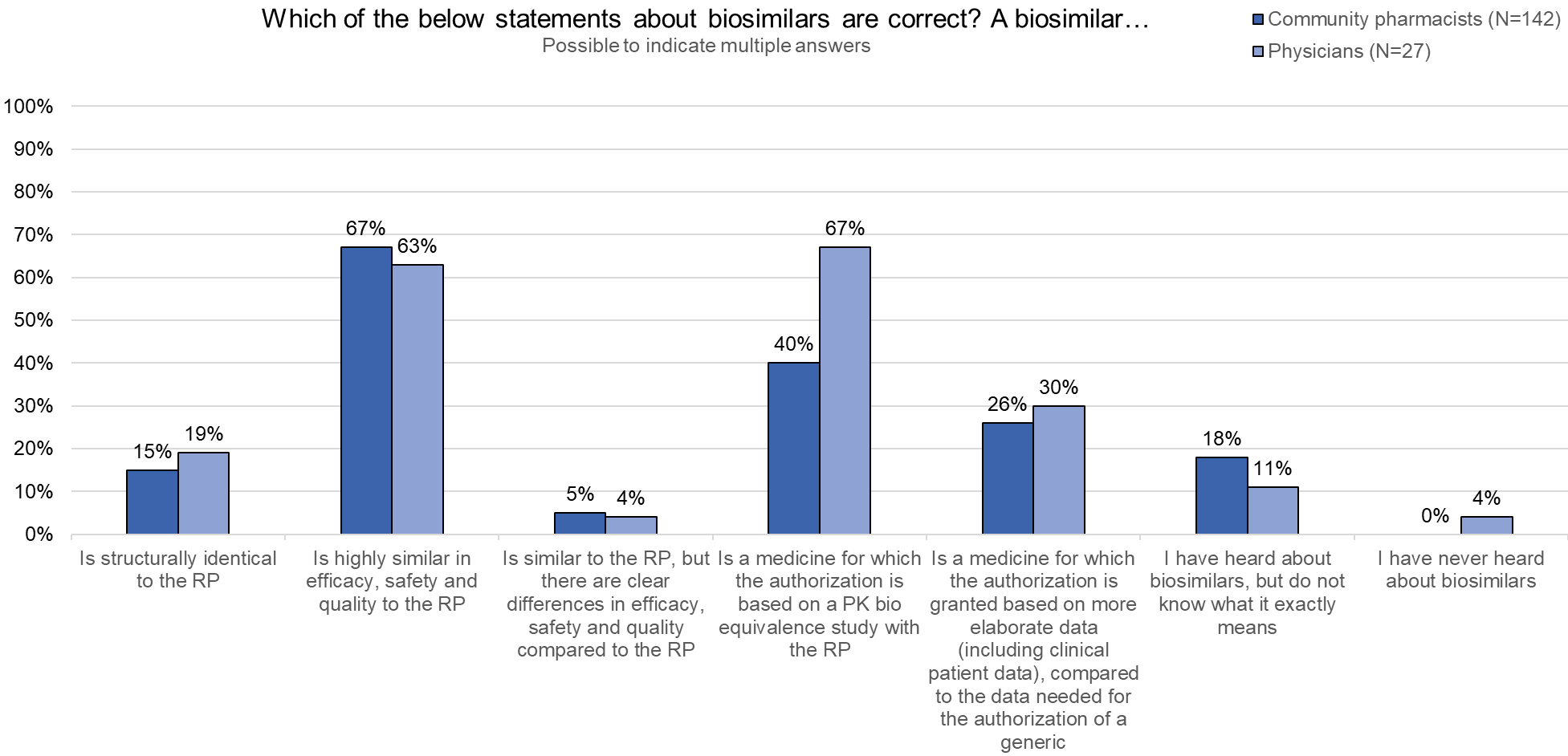
*N: number, PK: pharmacokinetic, RP: reference product*

Statistical testing: No statistical differences in knowledge between more recently graduated and more senior community pharmacists (more (N=60) *versus* less than 20 years (N=82) of pharmacy experience) were found (Table S3 in Supplementary Material)

**Figure S2.** Additional question on the self-assessed competence of community pharmacists to dispense biologicals (in general) and biosimilars (in particular)

*EUB: guidance upon first dispensing of a certain medicine, N: number, TUB: guidance upon second dispensing of a certain medicine*


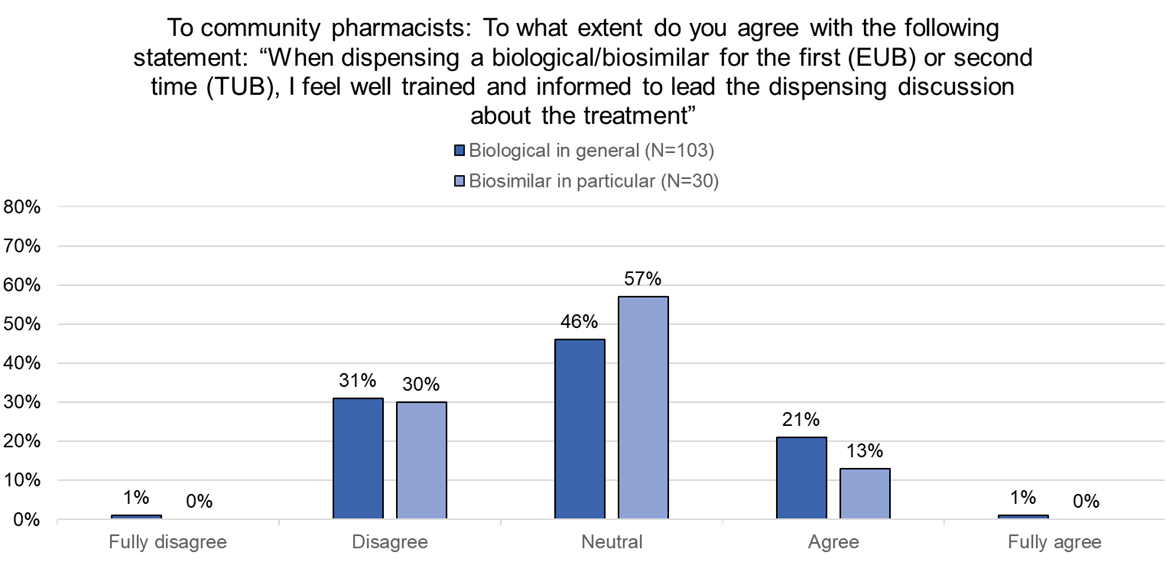


**Figure S3**. Opinion of community pharmacists about the organisation of a counselling treatment conversation for patient biosimilar use

*GGG: Begeleidingsgesprek Goed Gebruik Geneesmiddelen/Counseling treatment conversation correct use of medicines. A GGG aims to improve pharmaco-therapeutic care by means of counselling the patient with his or her medication use upon initiation of a new treatment. The initial information counselling is continued by a follow-up conversation when the treatment is renewed. In Belgium, the pharmacist receives a remuneration for such a GGG.*

*N: number, RP: reference product*


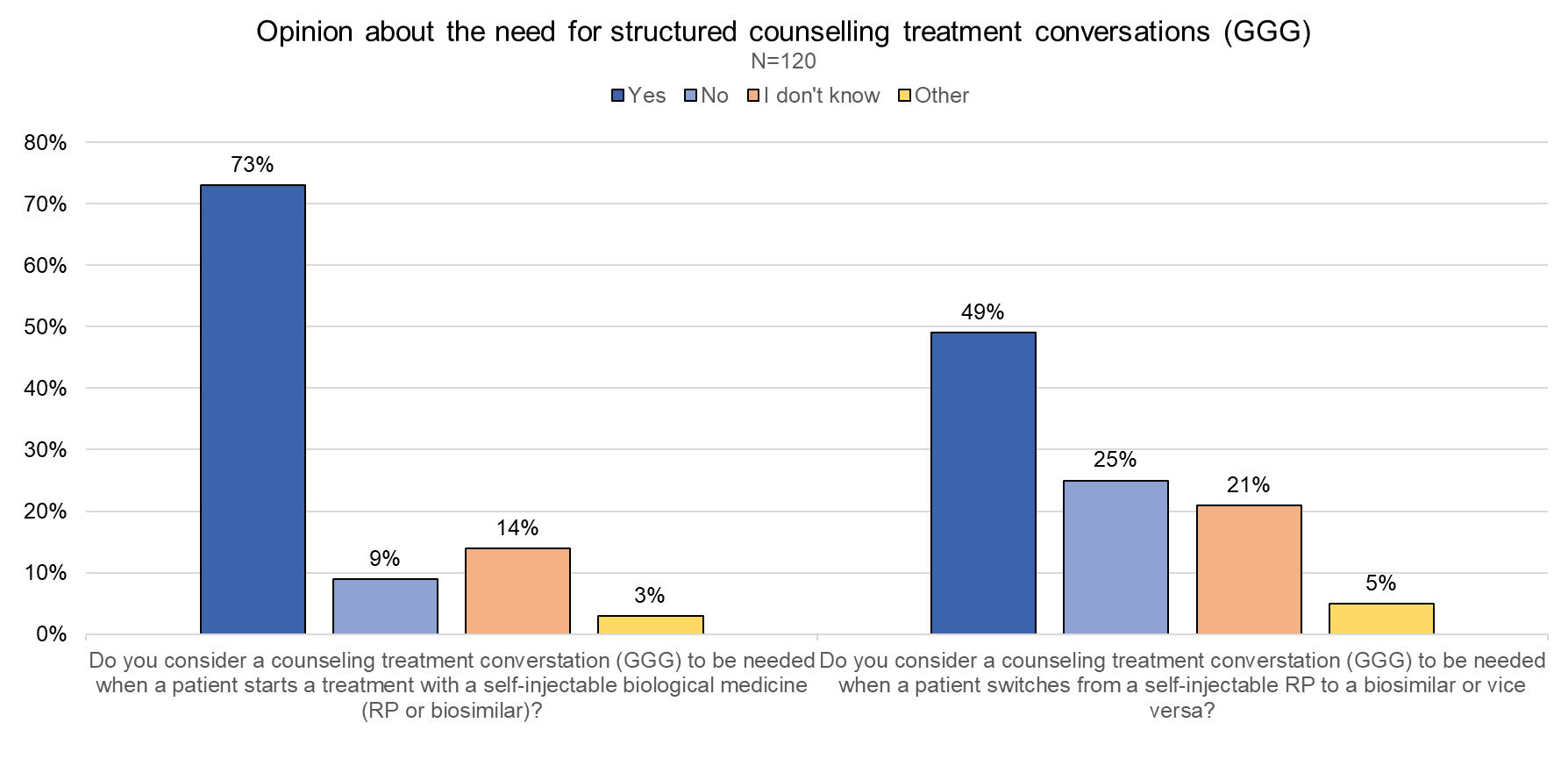


**Figure S4.** Additional questions posed to community pharmacists and physicians regarding interchangeability

*Interchangeability: interchangeability refers to the possibility of exchanging one medicine for another medicine that is expected to have the same clinical effect. This could mean replacing a reference product with a biosimilar (or vice versa) or replacing one biosimilar with another.*

*N: number, RP: reference product*


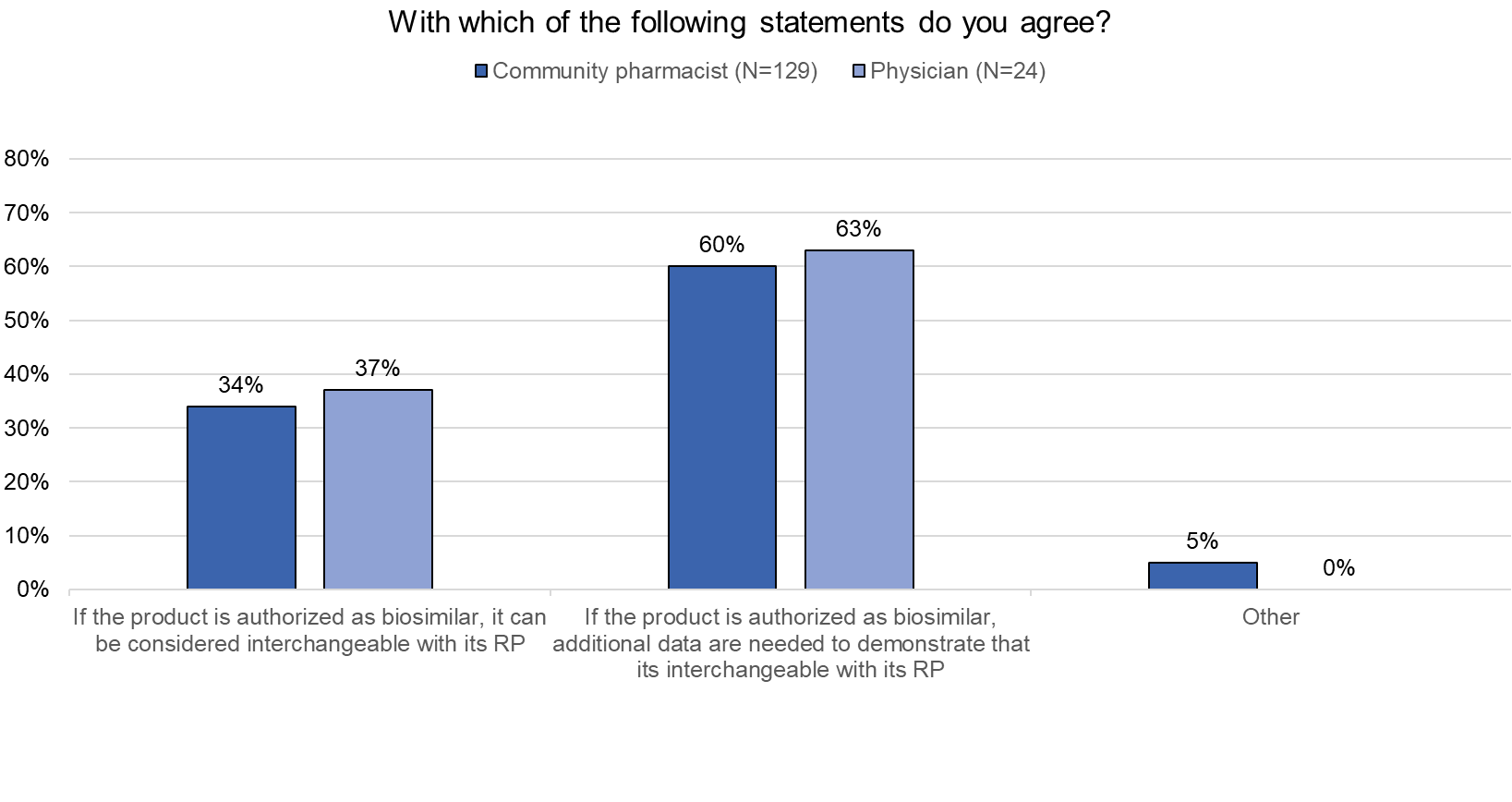
**a.**


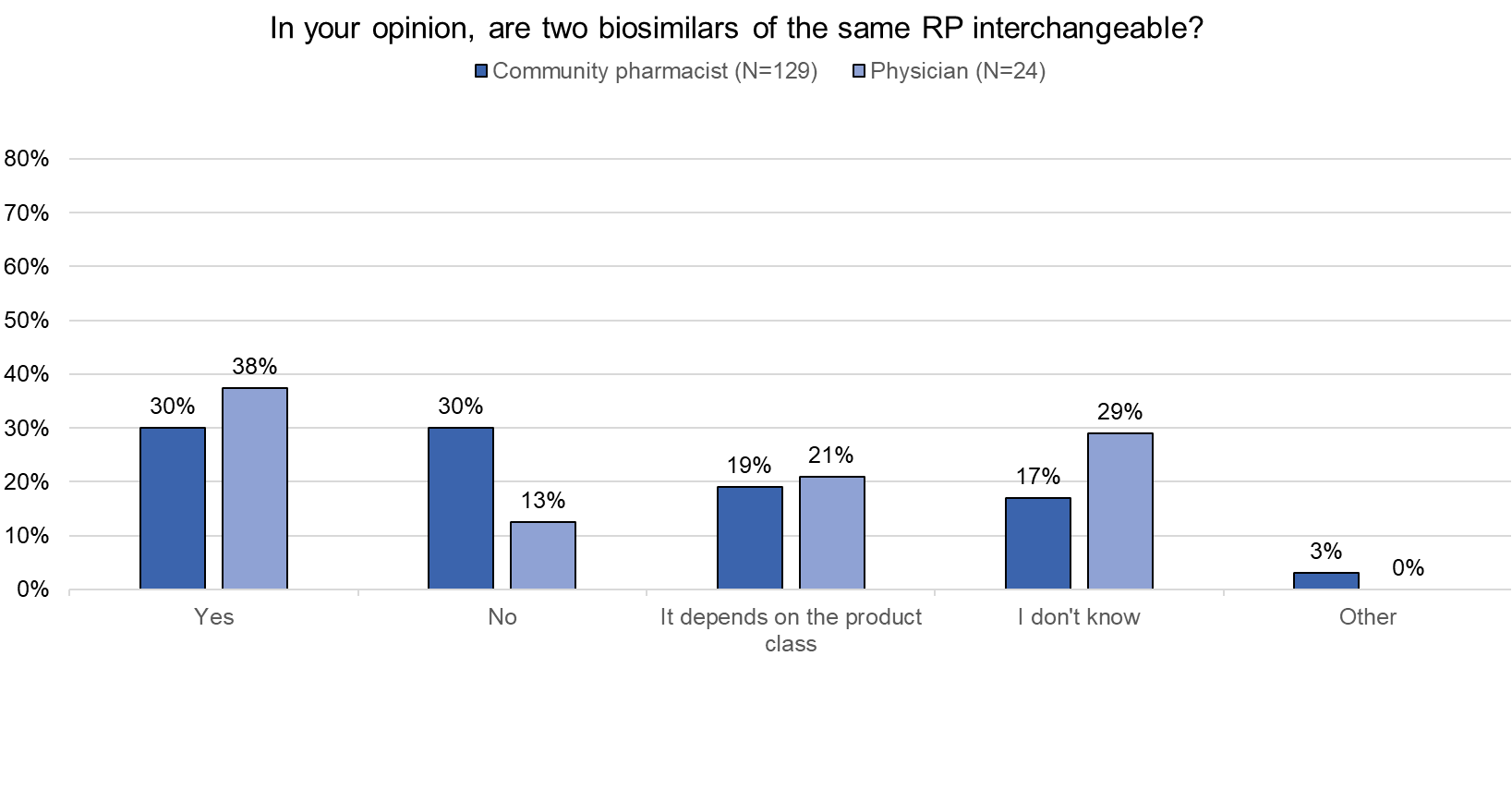
**b.**

**Figure S5** Additional questions posed to community pharmacists and physicians regarding substitution

*N: number, GP: general practitioner, RP: reference product*


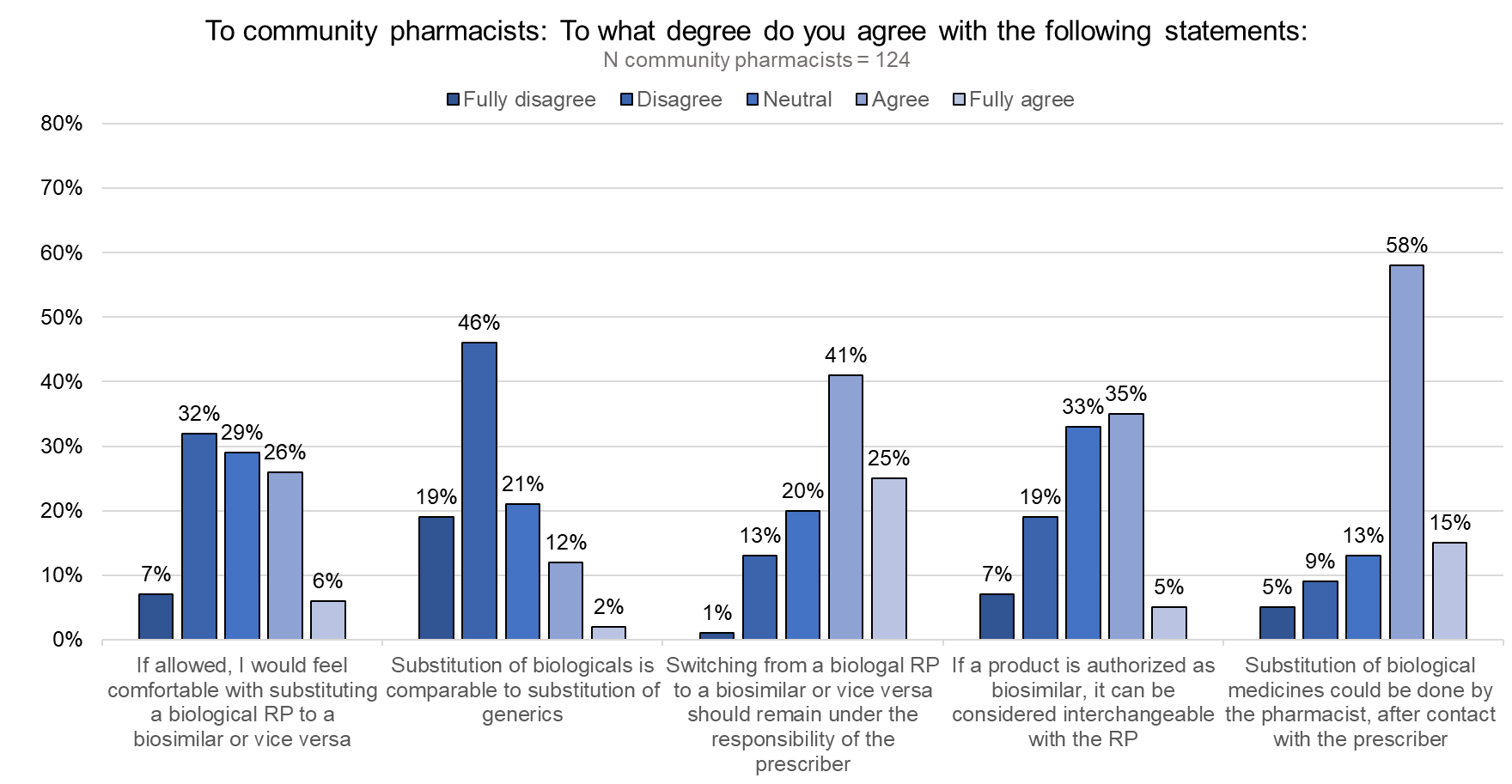
**a.**

**
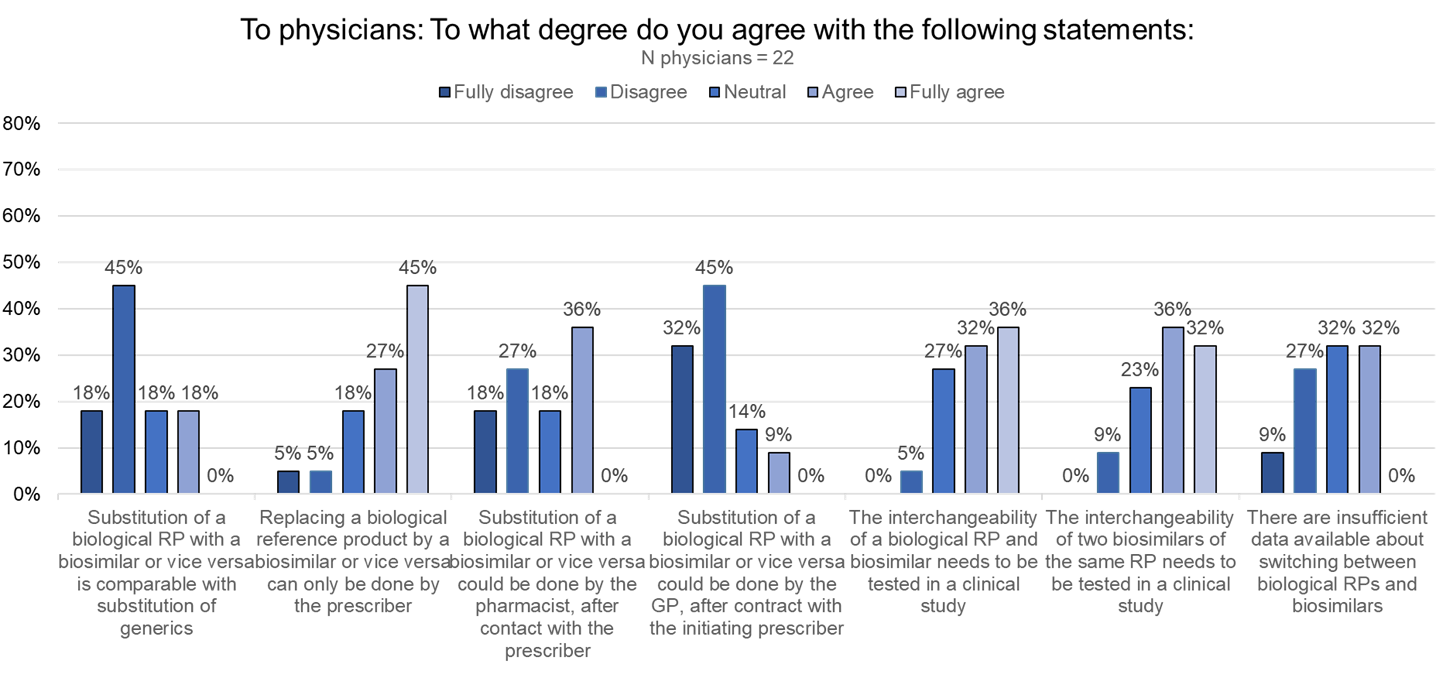
b.**

**Figure S6.** Additional questions posed to community pharmacists and physicians regarding information and training needs

*EMA: European Medicines Agency, FAMHP: Federal Agency for Medicines and Health Products (Belgian National Competent Authority), KOL: key opinion leader, LMWH: low molecular weight heparins, N: number, NIHDI: National Institute for Health and Disability Insurance (Belgian healthcare insurance agency), TNF-alfa blockers: tumor necrosis factor-alfa blockers*


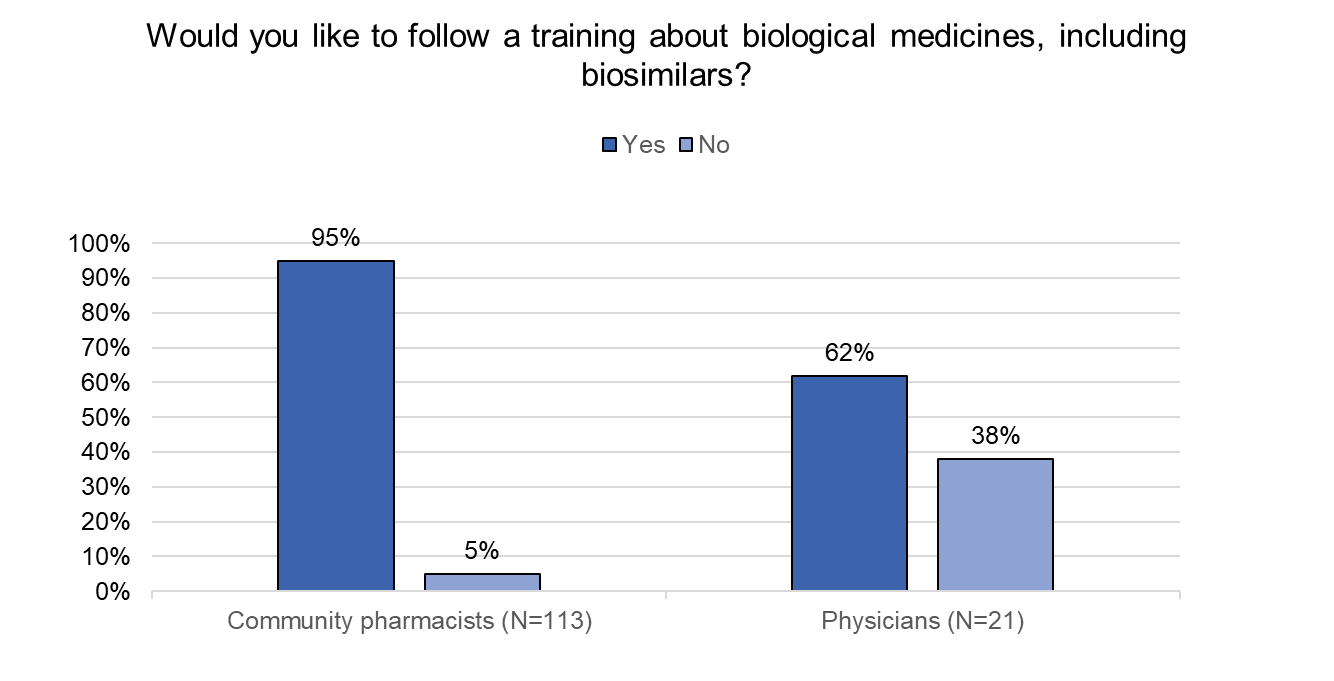
**a.**

**
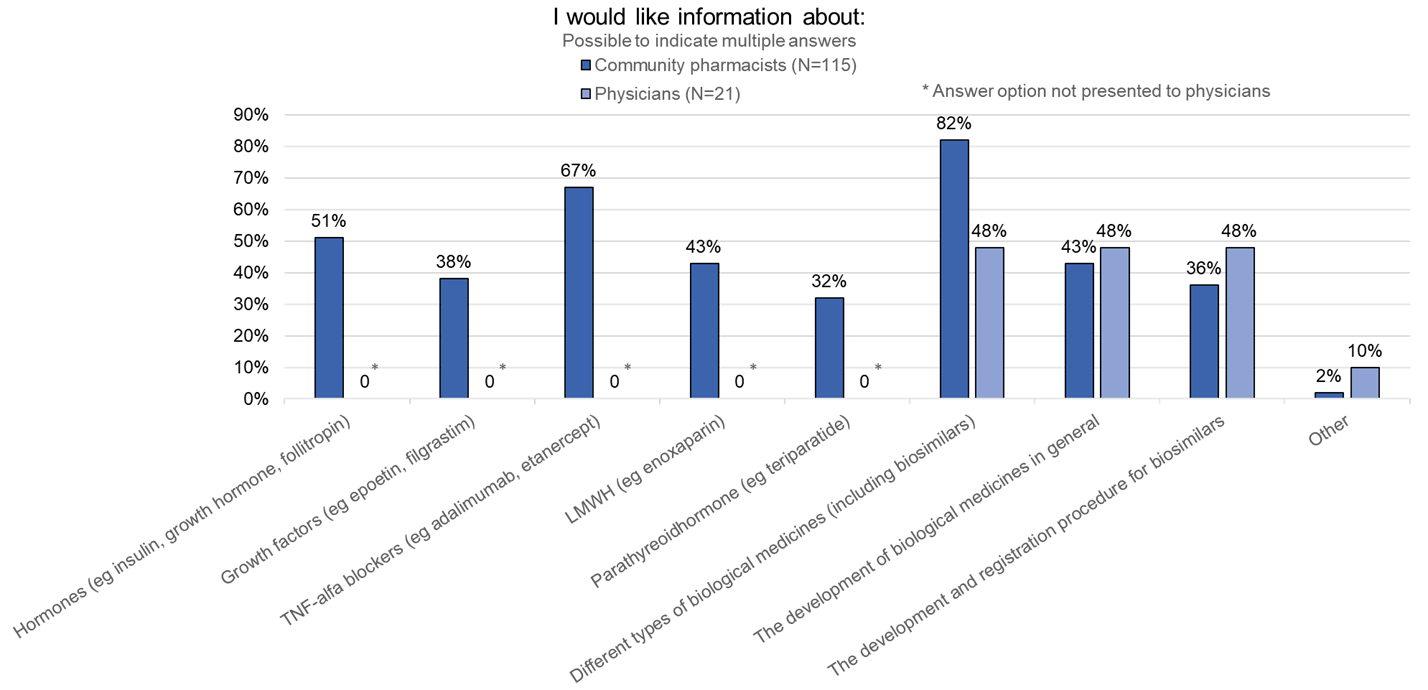
b.**

**c.**

**
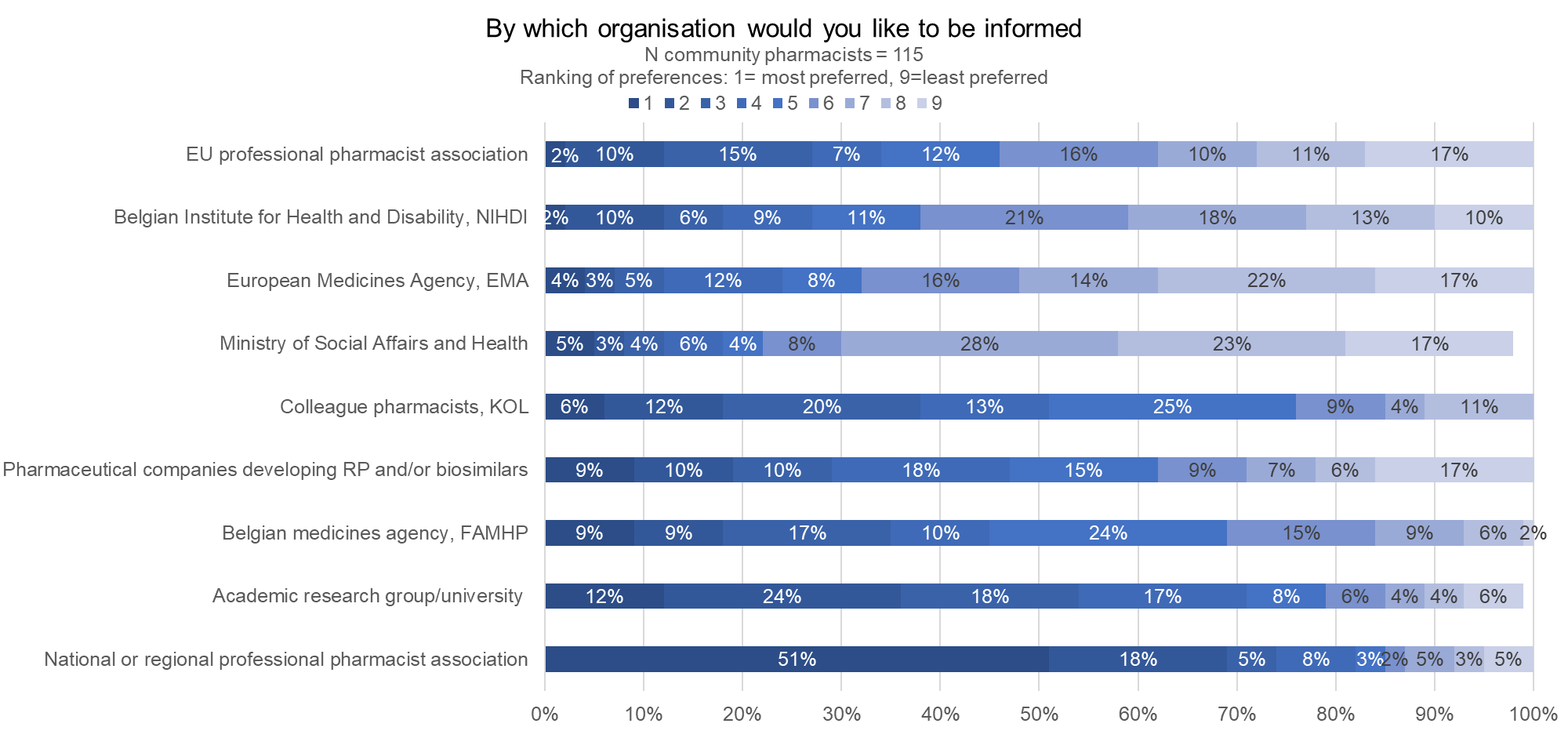
**

**
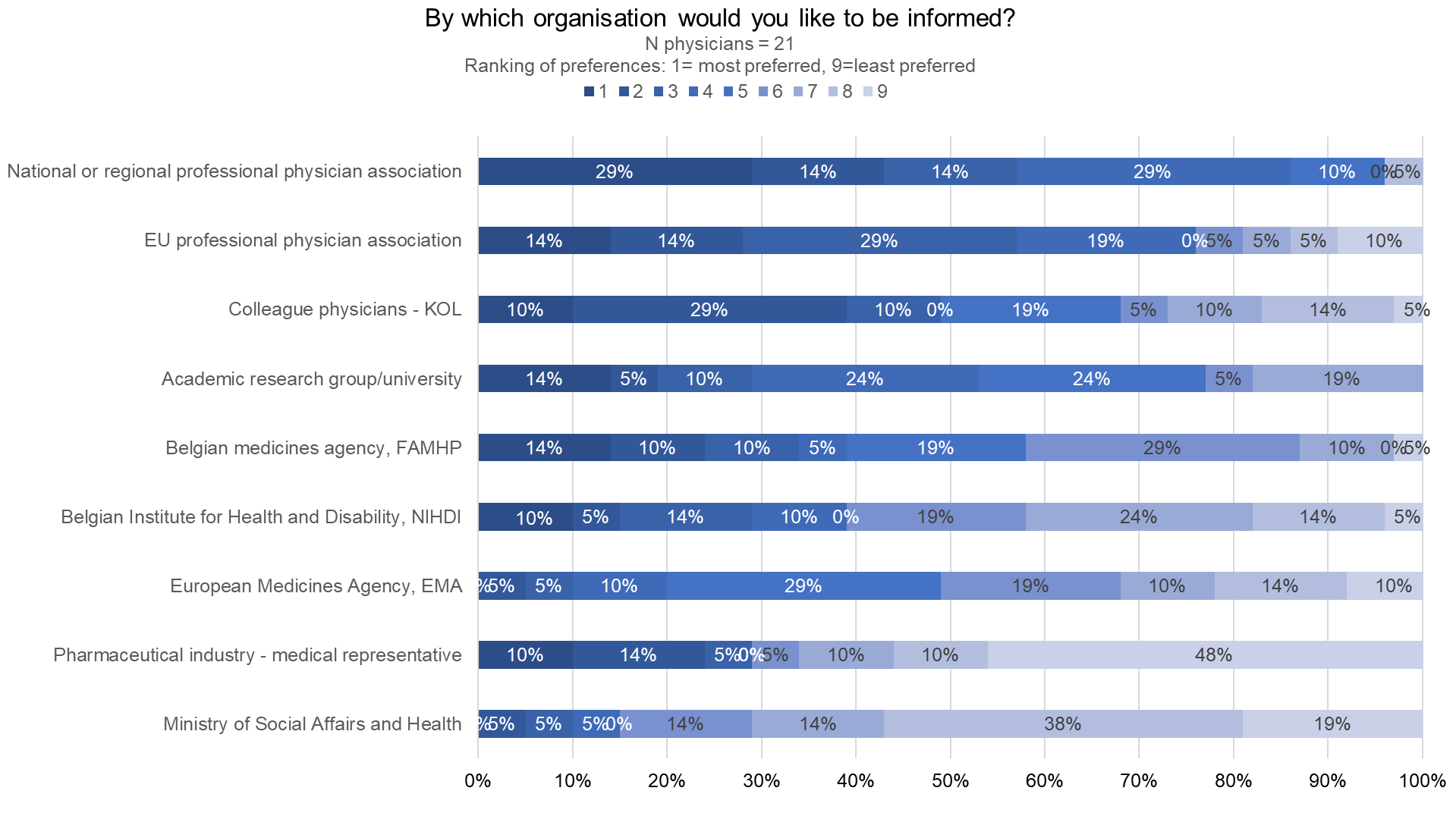
d.**

**e.**

**
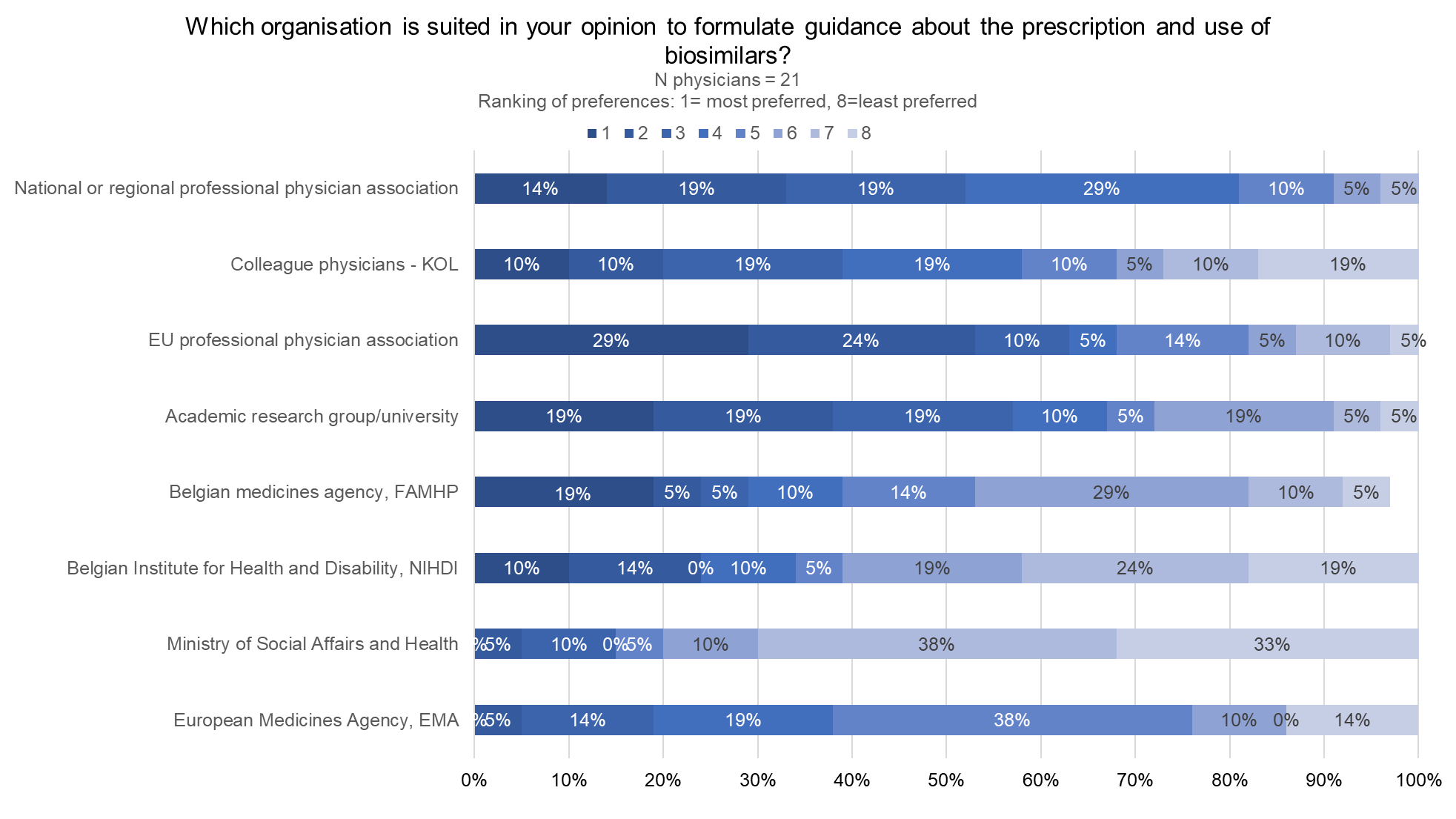
**

**Figure S7.** Reasons why physicians would not prescribe a biosimilar

*EMA: European Medicines Agency, N: number, RP: reference product*

*
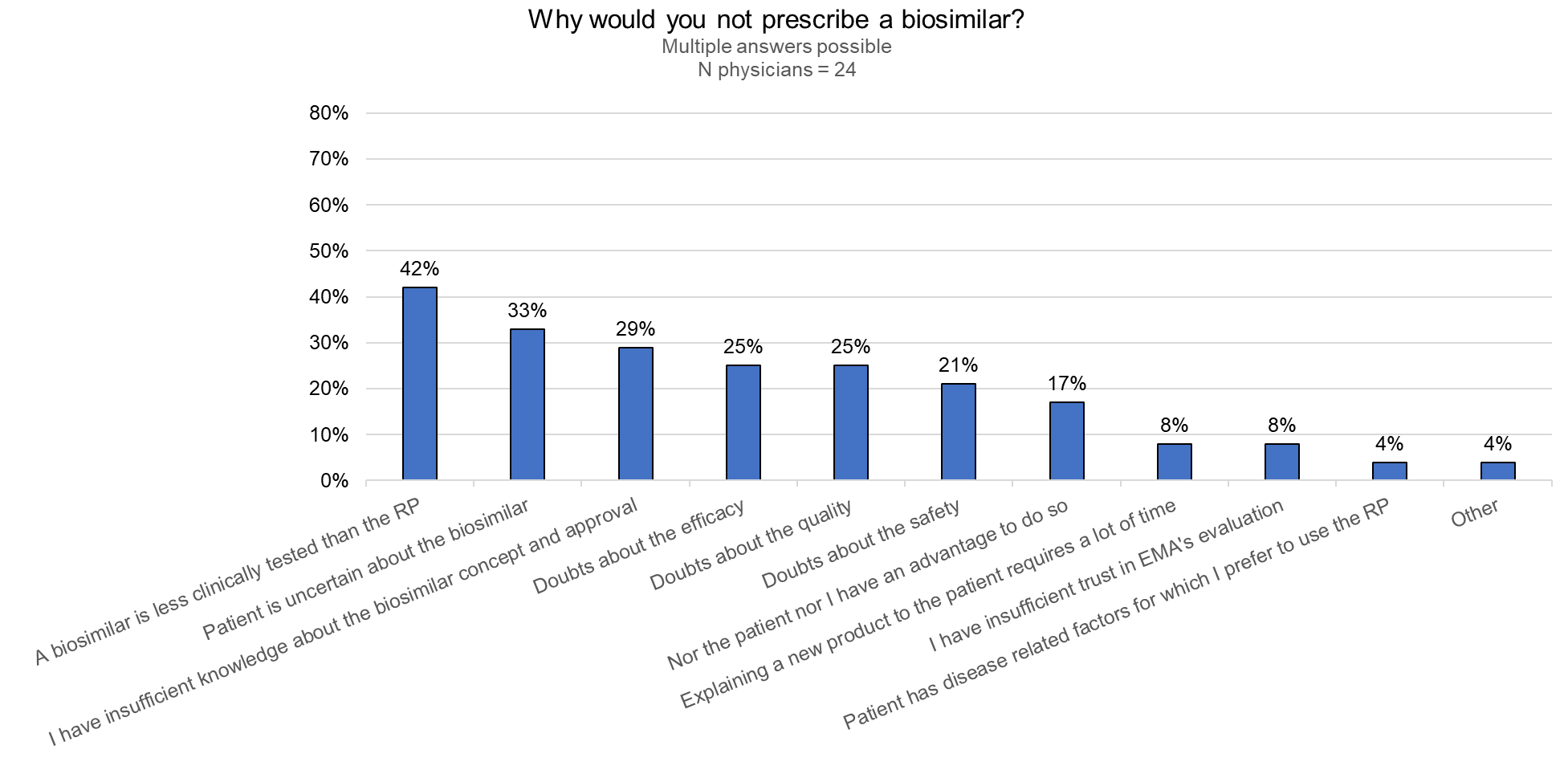
*

**Figure S8.** Questions about the need for incentives to stimulate biosimilar prescription in the ambulatory setting

*FAMHP: Federal Agency of Medicines and Health Products, N: number*

*
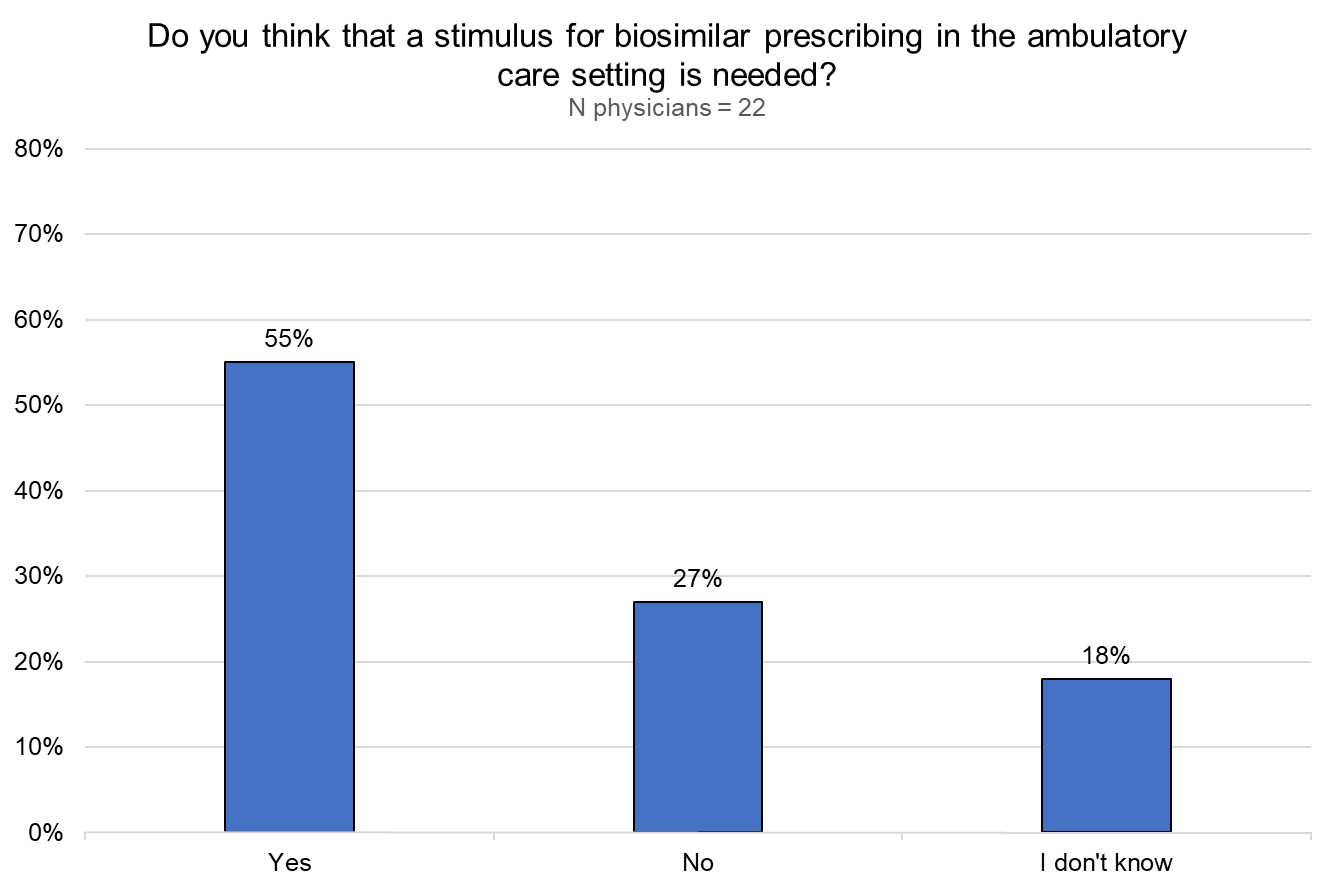
***a.**

*
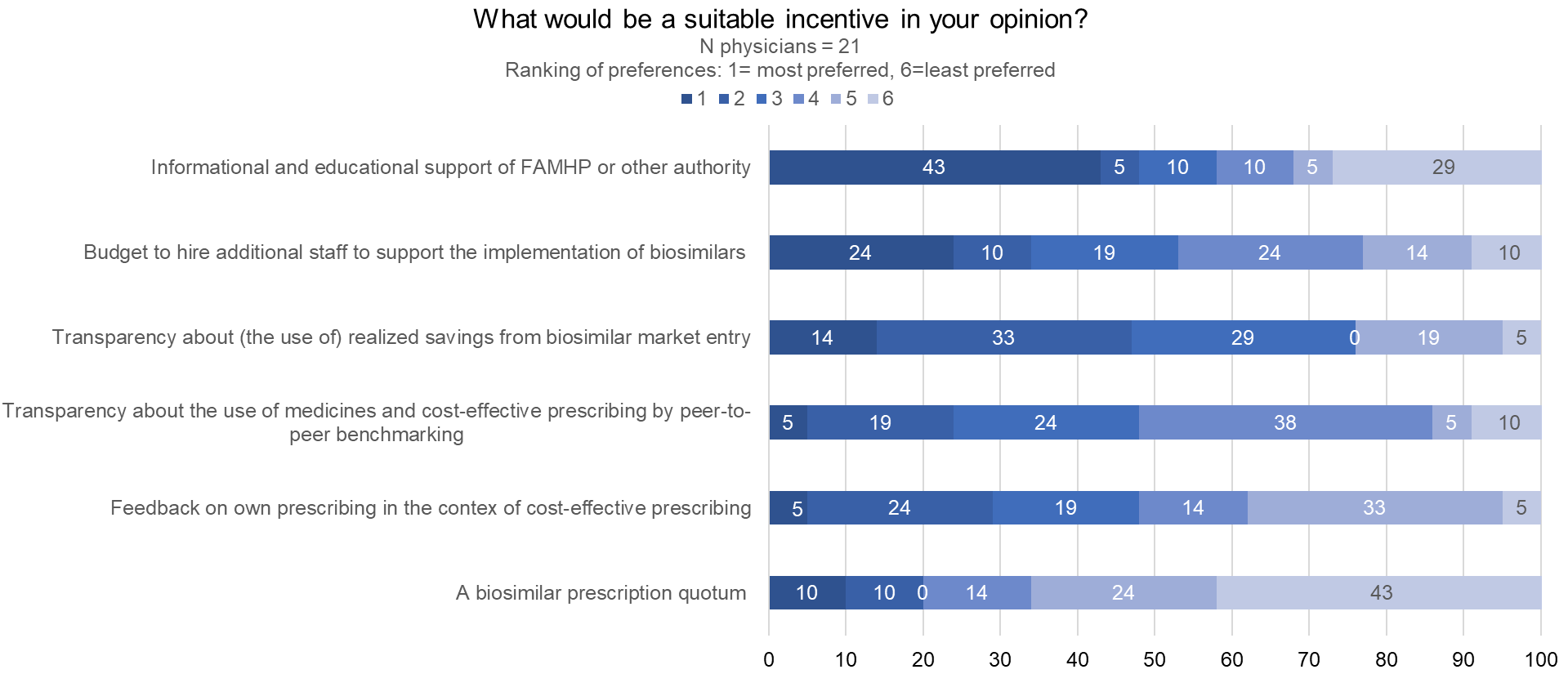
***b.**

**c.**

**
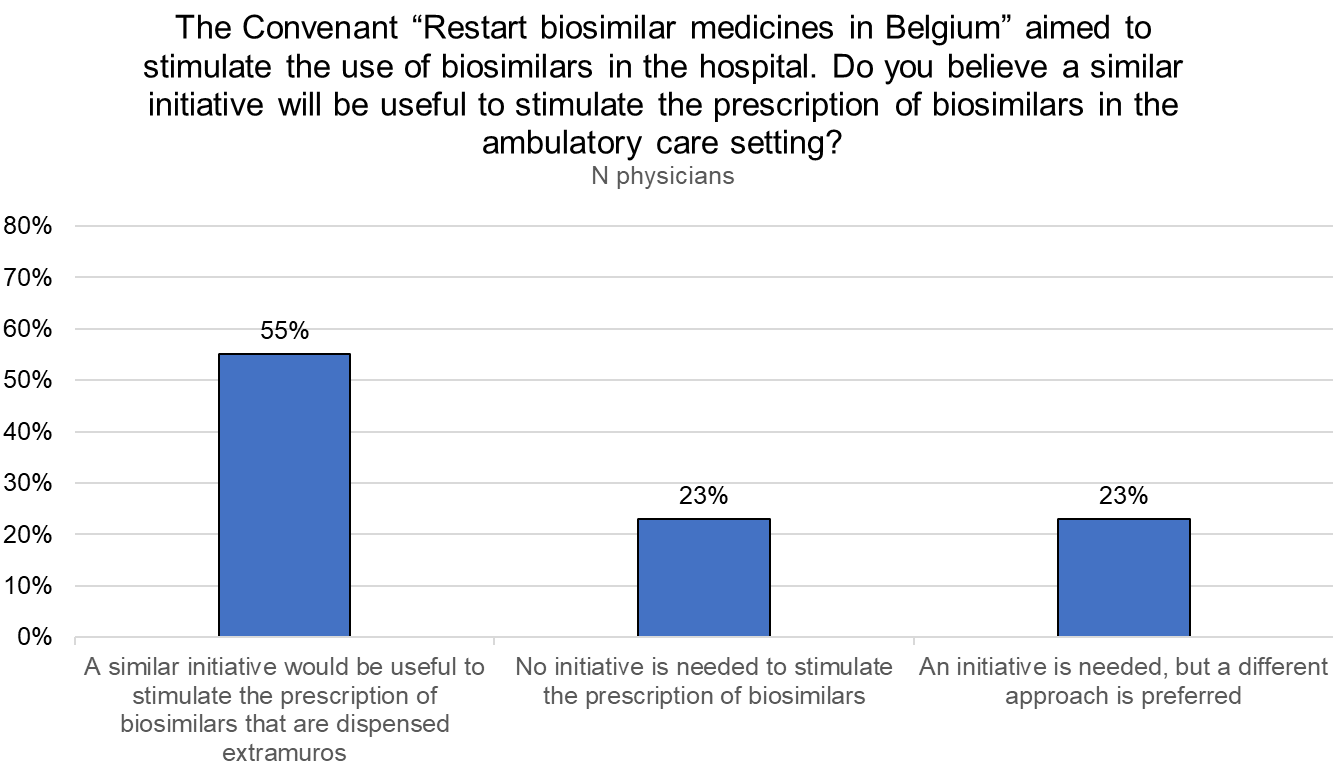
**
