# Supplementary material for: Knowledge and perception of biosimilars in ambulatory care: a survey among Belgian community pharmacists and physicians
Source: J Pharm Policy Pract. 2021 Jun 22;14:53. doi: 10.1186/s40545-021-00330-x (PMC8218462; doi:10.1186/s40545-021-00330-x)
Supplement: Supplementary file 2 — Additional file 2: Table S1. Community pharmacists: participants’ experience with biologicals in general. Table S2. Community pharmacists: participants’ experience with biosimilars. Table S3. Statistical analysis—testing for differences between experienced and more recently graduated pharmacists in terms of knowledge about biosimilars. Table S4. Statistical analysis—testing for differences between the self-assessed competences of pharmacists in dispensing biologicals in general versus biosimilars in particular. Table S5. Statistical analysis—testing for differences between the self-assessed competences in dispensing biologicals in general between experienced and more recently graduated pharmacists. Table S6. Statistical analysis—testing for differences between the self-assessed competences in dispensing biosimilars between experienced and more recently graduated pharmacists. Table S7. Physicians: participants’ experience with biologicals in general. Table S8. Physicians: participants’ experience with biosimilars. [file 40545_2021_330_MOESM2_ESM.docx]

**Knowledge and perception of biosimilars in ambulatory care: A survey among Belgian community pharmacists and physicians**

Liese Barbier, Yannick Vandenplas, Steven Simoens, Paul Declerck, Arnold G. Vulto, Isabelle Huys

Journal of Pharmaceutical Policy and Practice

Contact: liese.barbier@kuleuven.be

**Supplementary tables**

- **Table S1** Community pharmacists: participants’ experience with biologicals in general
- **Table S2** Community pharmacists: participants’ experience with biosimilars
- **Table S3** Statistical analysis – testing for differences between experienced and more recently graduated pharmacists in terms of knowledge about biosimilars
- **Table S4** Statistical analysis – testing for differences between the self-assessed competences of pharmacists in dispensing biologicals in general versus biosimilars in particular
- **Table S5** Statistical analysis – testing for differences between the self-assessed competences in dispensing biologicals in general between experienced and more recently graduated pharmacists
- **Table S6** Statistical analysis – testing for differences between the self-assessed competences in dispensing biosimilars between experienced and more recently graduated pharmacists
- **Table S7** Physicians: participants’ experience with biologicals in general
- **Table S8** Physicians: participants’ experience with biosimilars

| **Table S1 Community pharmacists: participants’ experience with biologicals in general** | | | |
| --- | --- | --- | --- |
| Experience with biologicals | Community pharmacists | | n = |
|  | n | % |  |
| Have you dispensed a biological medicine? |  |  | 177 |
| Yes | 148 | 84 |  |
| No | 7 | 4 |  |
| I don’t know | 22 | 12 |  |
| What is your experience with dispensing biological medicines? |  |  | 125 |
| I dispense different types of biologicals such as insulin, heparine, TNF-alfa blockers, on a daily or weekly basis | 69 | 55 |  |
| I dispense biologicals such as insulin and heparin on a daily or weekly basis and dispense biological such as TNF-alfa blockers on a monthly basis | 45 | 36 |  |
| I dispense biologicals such as insulin and heparin on a daily or weekly basis but do not or rarely dispense biological such as TNF-alfa blockers | 7 | 6 |  |
| I do not dispense biologicals on a recurrent basis (monthly, weekly, daily) | 3 | 2 |  |
| Other | 0 | 0 |  |
| With which classes of biological medicines do you have experience dispensing? |  |  | 125 |
| Hormones (eg. insulin, GH, follitropin) | 118 | 94 |  |
| Growth factors (eg epo, filgrastim) | 28 | 22 |  |
| TNF-alfa blokkers (eg adalimumab, etanercept) | 119 | 95 |  |
| LMWH (eg enoxaparin) | 11 | 89 |  |
| Parathyreoidhormone (eg teriparatide) | 6 | 5 |  |
| I don’t know | 1 | 1 |  |
| Other | 1 | 1 |  |
| How many of your patients are treated with a biological? |  |  | 125 |
| ≤ 10% | 65 | 52 |  |
| > 10% – 20% | 38 | 30 |  |
| > 20 – 30% | 3 | 2 |  |
| > 30 – 50% | 9 | 7 |  |
| > 50 – 70% | 1 | 1 |  |
| > 70% | 0 | 0 |  |
| I don’t know | 9 | 7 |  |
| Have you followed a training or symposium about biological medicines? |  |  | 113 |
| Yes | 47 | 42 |  |
| No | 66 | 58 |  |
| *GH: growth hormone, LMWH: low molecular weight heparin, N: number, TNF: tumor necrosis factor,*  *Percentages are rounded to the nearest integer* | | | |

| **Table S2 Community pharmacists: participants’ experience with biosimilars** | | | |
| --- | --- | --- | --- |
| Experience with biosimilars | Community pharmacists | | n = |
|  | n | % |  |
| Have you dispensed a biosimilar? |  |  | 139 |
| Yes | 45 | 32 |  |
| No | 50 | 36 |  |
| I don’t know | 45 | 32 |  |
| With which classes of biosimilar medicines do you have experience dispensing? |  |  | 36 |
| Hormones (eg. insulin, GH, follitropin) | 24 | 67 |  |
| Growth factors (eg epo, filgrastim) | 2 | 6 |  |
| TNF-alfa blokkers (eg adalimumab, etanercept) | 23 | 64 |  |
| LMWH (eg enoxaparin) | 12 | 33 |  |
| Parathyreoidhormone (eg teriparatide) | 0 | 0 |  |
| I don’t know | 3 | 8 |  |
| Other | 0 | 0 |  |
| How many of your patients are treated with a biosimilar? |  |  | 36 |
| 0% | 1 | 3 |  |
| ≤ 10% | 27 | 75 |  |
| > 10% – 20% | 2 | 6 |  |
| > 20 – 30% | 1 | 3 |  |
| > 30 – 50% | 1 | 3 |  |
| > 50 – 70% | 0 | 0 |  |
| > 70% | 0 | 0 |  |
| I don’t know | 4 | 11 |  |
| Have you followed a training or symposium about biosimilars? |  |  | 113 |
| Yes | 25 | 22 |  |
| No | 88 | 78 |  |
| *GH: growth hormone, LMWH: low molecular weight heparin, N: number, TNF: tumor necrosis factor,*  *Percentages are rounded to the nearest integer* | | | |

For the statistical analysis (as shown in Tables S3-S6), a Fisher Exact test (two tailed) was conducted. The Fisher Exact test is standardly used (standard practice, unless computational restrictions require the use of a Chi² test) to compare proportions of categorical data, and may be especially best suited for smaller samples.^[[1]](#footnote-1)^

| **Table S3 Statistical analysis – testing for differences between experienced and more recently graduated pharmacists* in terms of knowledge about biosimilars** | |
| --- | --- |
| **Question: Which of the below statements about biosimilars are correct? A biosimilar …** | |
|  | **Fisher Exact test**  **(two tailed)** |
|  | **p-value** |
| **Statement 1:**  Is structural identical to the RP | p=,81603 |
| **Statement 2:**  Is highly similar in efficacy, safety, and quality to the RP | p=,07286 |
| **Statement 3:**  Is similar to the RP, but there are clear differences in efficacy, safety, and quality compared to the RP | p=1,0000 |
| **Statement 4:**  Is a medicine for which the authorization is based on a PK bio equivalence study with the RP | p=,73195 |
| **Statement 5:**  Is a medicine for which the authorization is granted based on more elaborate clinical data compared to the data needed for the authorization of a generic | p=,33882 |
| **Statement 6:**  I have heard about biosimilars, but do not know what it exactly means | p=,07295 |
| *Answers of respondents with more (N=60) *versus* less than 20 years (N=82) of pharmacy experience were compared | |
| *RP: reference product* | |

| **Table S4**  **Statistical analysis – testing for differences between the self-assessed competences of pharmacists in dispensing biologicals in general *versus* biosimilars in particular** | |
| --- | --- |
| **Question: When I dispense a biological/biosimilar, I feel …** | |
|  | **Fisher Exact test**  **(two tailed)** |
|  | **p-value** |
| **Statement 1:**  I feel well trained and informed to dispense and guide patients in their treatment with this type of medicine | p=1,0000 |
| **Statement 2:**  I feel insufficiently trained and informed to dispense and guide patients in their treatment with this type of medicine | **p=,02283** |
| **Statement 3:**  I feel well trained and informed to answer questions from patients about this treatment | p=,62365 |
| **Statement 4:**  I feel unsufficiently trained and informed to answer questions from patients about this treatment | p=,48461 |
| **Statement 5:**  I feel a need for more education or information about this type of medicine | p=,84271 |
| **Statement 6:**  I feel comfortable with dispensing not all, but some less complex products | p=,33908 |
| *Answers for biologicals in general (N=125) *versus* biosimilars in general (N=36) were compared | |

| **Table S5 Statistical analysis – testing for differences between the self-assessed competences in dispensing biologicals in general between experienced and more recently graduated pharmacists*** | |
| --- | --- |
| **Question: When I dispense a biological, I feel …** | |
|  | **Fisher Exact test**  **(two tailed)** |
|  | **p-value** |
| **Statement 1:**  I feel well trained and informed to dispense and guide patients in their treatment with this type of medicine | **p=,03210** |
| **Statement 2:**  I feel insufficiently trained and informed to dispense and guide patients in their treatment with this type of medicine | p=,80967 |
| **Statement 3:**  I feel well trained and informed to answer questions from patients about this treatment | p=,80621 |
| **Statement 4:**  I feel insufficiently trained and informed to answer questions from patients about this treatment | p=,16970 |
| **Statement 5:**  I feel a need for more education or information about this type of medicine | p=,33103 |
| **Statement 6:**  I feel comfortable with dispensing not all, but some less complex products | p=,71040 |
| *Answers of respondents with more (N=47) *versus* less than 20 years (N=78) of pharmacy experience were compared | |

| **Table S6 Statistical analysis – testing for differences between the self-assessed competences in dispensing biosimilars between experienced and more recently graduated pharmacists*** | |
| --- | --- |
| **Question: When I dispense a biosimilar, I feel …** | |
|  | **Fisher Exact test**  **(two tailed)** |
|  | **p-value** |
| **Statement 1:**  I feel well trained and informed to dispense and guide patients in their treatment with this type of medicine | p=1,0000 |
| **Statement 2:**  I feel unsufficiently trained and informed to dispense and guide patients in their treatment with this type of medicine | p=,73013 |
| **Statement 3:**  I feel well trained and informed to answer questions from patients about this treatment | p=,22848 |
| **Statement 4:**  I feel unsufficiently trained and informed to answer questions from patients about this treatment | p=1,0000 |
| **Statement 5:**  I feel a need for more education or information about this type of medicine | p=,72055 |
| **Statement 6:**  I feel comfortable with dispensing not all, but some less complex products | p=,30195 |
| *Answers of respondents with more (N=17) *versus* less than 20 years (N=19) of pharmacy experience were compared | |

| **Table S7 Physicians: participants’ experience with biologicals in general** | | | |
| --- | --- | --- | --- |
| Experience with biologicals | Physicians | | n = |
|  | n | % |  |
| Do you prescribe biological medicines? |  |  | 30 |
| Yes | 23 | 77 |  |
| No, I haven’t (yet) prescribed a biological, but I follow patients under treatment with a biological | 2 | 7 |  |
| No, I haven’t (yet) prescribed a biological and I do not follow patients under treatment with a biological | 2 | 7 |  |
| I don’t know | 3 | 10 |  |
| With which classes of biological medicines do you have experience in practice (prescribing or follow-up)? |  |  | 23 |
| Hormones (eg. insulin, GH, follitropin) | 6 | 26 |  |
| Growth factors (eg epo, filgrastim) | 3 | 13 |  |
| TNF-alfa blokkers (eg adalimumab, etanercept) | 19 | 83 |  |
| Other monoclonal antibodies (eg rituximab) | 14 | 61 |  |
| LMWH (eg enoxaparin) | 11 | 48 |  |
| Parathyreoidhormone (eg teriparatide) | 6 | 26 |  |
| I don’t know | 0 | 0 |  |
| Other | 2 | 9 |  |
| How many of your patients are treated with a biological? |  |  | 23 |
| ≤ 10% | 0 | 0 |  |
| > 10% – 20% | 11 | 48 |  |
| > 20 – 30% | 6 | 26 |  |
| > 30 – 50% | 5 | 22 |  |
| > 50 – 70% | 1 | 4 |  |
| > 70% | 0 | 0 |  |
| I don’t know | 0 | 0 |  |
| Have you followed a training or symposium about biological medicines? |  |  | 21 |
| Yes | 15 | 71 |  |
| No | 6 | 29 |  |
| *GH: growth hormone, LMWH: low molecular weight heparin, N: number, TNF: tumor necrosis factor,*  *Percentages are rounded to the nearest integer* | | | |

| **Table S8 Physicians: participants’ experience with biosimilars** | | | |
| --- | --- | --- | --- |
| Experience with biosimilars | Physicians | | n = |
|  | n | % |  |
| Have you prescribed a biosimilar (as initiation or continuation of treatment)? |  |  | 27 |
| Yes | 14 | 52 |  |
| No, I haven’t (yet) prescribed a biosimilar, but I follow a patient(s) under treatment with a biosimilar | 2 | 7 |  |
| No, I have no experience with patients under treatment of biosimilars | 7 | 26 |  |
| I don’t know | 4 | 15 |  |
| With which classes of biosimilar medicines do you have experience in practice (prescribing or follow-up)? |  |  | 16 |
| Hormones (eg. insulin, GH, follitropin) | 3 | 19 |  |
| Growth factors (eg epo, filgrastim) | 1 | 6 |  |
| TNF-alfa blokkers (eg adalimumab, etanercept) | 12 | 75 |  |
| Other monoclonal antibodies (eg rituximab) | 3 | 19 |  |
| LMWH (eg enoxaparin) | 2 | 12,5 |  |
| Parathyreoidhormone (eg teriparatide) | 0 | 0 |  |
| I don’t know | 0 | 0 |  |
| Other | 0 | 0 |  |
| How many of your patients are treated with a biosimilar? |  |  | 16 |
| 0% | 0 | 0 |  |
| ≤ 10% | 13 | 81 |  |
| > 10% – 20% | 0 | 0 |  |
| > 20 – 30% | 3 | 19 |  |
| > 30 – 50% | 0 | 0 |  |
| > 50 – 70% | 0 | 0 |  |
| > 70% | 0 | 0 |  |
| I don’t know | 0 | 0 |  |
| Have you followed a training or symposium about biosimilars? |  |  | 21 |
| Yes | 11 | 52 |  |
| No | 10 | 48 |  |
| Do you have experience with switching a patient under treatment with a reference product to a biosimilar? |  |  | 24 |
| Yes | 8 | 33 |  |
| No | 16 | 67 |  |
| *GH: growth hormone, LMWH: low molecular weight heparin, N: number, TNF: tumor necrosis factor,*  *Percentages are rounded to the nearest integer* | | | |

1. Geert Verbeke. *Pharmaceutical data analysis*. 2020 [↑](#footnote-ref-1)
