# Supplementary material for: Knowledge and perception of biosimilars in ambulatory care: a survey among Belgian community pharmacists and physicians
Source: J Pharm Policy Pract. 2021 Jun 22;14:53. doi: 10.1186/s40545-021-00330-x (PMC8218462; doi:10.1186/s40545-021-00330-x)
Supplement: Supplementary file 3 — Additional file 3: Supplementary Box 1 Key terminology. [file 40545_2021_330_MOESM3_ESM.docx]

**Knowledge and perception of biosimilars in ambulatory care: A survey among Belgian community pharmacists and physicians**

Liese Barbier, Yannick Vandenplas, Steven Simoens, Paul Declerck, Arnold G. Vulto, Isabelle Huys

Journal of Pharmaceutical Policy and Practice

Contact: liese.barbier@kuleuven.be

**Supplementary Box 1** Key terminology

| **Box 1. Key terminology** | |
| --- | --- |
| A biological medicine | A medicine whose active substance is produced by or extracted from a biological source such as living cells or organisms (1). In contrast to chemically synthesized product, biologicals are generally large and complex mixtures, which may be less easy to fully characterize (2). |
| A biosimilar | A biological medicine that is similar to another, already approved original biological product (the reference biological medicine) (3). A biosimilar has a demonstrated similarity in physicochemical characteristics, efficacy and safety, based on a comprehensive comparability exercise with the reference biological medicine (4),(5). |
| A small molecule medicine | A traditional chemical drug, i.e. a small, low molecular weight chemically synthesized medicine (1),(2). |
| A generic | A copy of an already approved chemically synthesized medicine (1),(2). |
| Biosimilarity | The “demonstration of high similarity to a reference biological medicine in terms of chemical structure, biological activity and efficacy, safety and immunogenicity profile, mainly based on comprehensive comparability studies.” (5) |
| Interchangeability | “Refers to the possibility of exchanging one medicine for another medicine that is expected to have the same clinical effect. This could mean replacing a reference product with a biosimilar (or vice versa) or replacing one biosimilar with another.” (5) |
| Switching | “When the prescriber decides to exchange one medicine for another medicine with the same therapeutic intent.” (5) |
| Substitution | “The practice of dispensing one medicine instead of another equivalent and interchangeable medicine at pharmacy level without consulting the prescriber.” (5) |

1. Declerck P, Danesi R, Petersel D, Jacobs I. The Language of Biosimilars: Clarification, Definitions, and Regulatory Aspects. Drugs. 2017;77(6):671–7.

2. US Food & Drug Administration. What Are “Biologics” Questions and Answers [Internet]. [cited 2021 May 14]. Available from: https://www.fda.gov/about-fda/center-biologics-evaluation-and-research-cber/what-are-biologics-questions-and-answers

3. European Medicines Agency. Guideline on similar biological medicinal products. 2014.

4. Weise M, Bielsky M-C, De Smet K, Ehmann F, Ekman N, Narayanan G, et al. Biosimilars—why terminology matters. Nat Biotechnol. 2011;29(8):690–3.

5. European Medicines Agency. Biosimilars in the EU - Information guide for healthcare professionals. 2017.
